# Improved simplified clinical algorithm for identifying patients eligible for immediate initiation of antiretroviral therapy for HIV (SLATE II): protocol for a randomized evaluation

**DOI:** 10.1186/s13063-018-2928-5

**Published:** 2018-10-11

**Authors:** S Rosen, M Maskew, A T Brennan, M P Fox, L Vezi, P D Ehrenkranz, W D F Venter

**Affiliations:** 1 Department of Global Health, Boston University School of Public Health, 801 Massachusetts Ave Room 390, Boston, MA 02118 USA; 2 Health Economics and Epidemiology Research Office, Department of Internal Medicine, School of Clinical Medicine, Faculty of Health Sciences, University of the Witwatersrand, Johannesburg, South Africa; 3 Department of Epidemiology, Boston University School of Public Health, Boston, MA USA; 4 Bill & Melinda Gates Foundation, Seattle, WA USA; 5 Wits Reproductive Health and HIV Institute, Department of Internal Medicine, School of Clinical Medicine, Faculty of Health Sciences, University of the Witwatersrand, Johannesburg, South Africa

**Keywords:** Antiretroviral therapy, Same-day, Treatment initiation, Africa, South Africa, Protocol, Randomized trial

## Abstract

**Background:**

The World Health Organization recommends rapid (≤ 7 days) or same-day initiation of antiretroviral treatment (ART) for HIV-positive patients. South Africa adopted this recommendation in 2017, but multiple clinic visits, long waiting times, and delays for laboratory tests remain common. Streamlined approaches to same-day initiation that allow the majority of patients to start ART immediately, while ensuring that patients who do require additional services receive them, are needed to achieve national and international treatment program goals.

**Methods/Design:**

The SLATE II (Simplified Algorithm for Treatment Eligibility) study is an individually randomized evaluation of a clinical algorithm to reliably determine a patient’s eligibility for immediate ART initiation without waiting for laboratory results or additional clinic visits. It differs from the earlier SLATE I study in management of patients with symptoms of tuberculosis (under SLATE II these patients may be started on ART immediately) and other criteria for immediate initiation. SLATE II will randomize (1:1) 600 adult, HIV-positive patients who present for HIV testing or care and are not yet on ART in South Africa. Patients randomized to the standard arm will receive standard-of-care ART initiation from clinic staff. Patients randomized to the intervention arm will be administered a symptom report, medical history, brief physical exam, and readiness assessment. Symptomatic patients will also have a tuberculosis (TB) module with lipoarabinomannan antigen of mycobacteria test. Patients who have satisfactory results for all four components will be dispensed antiretrovirals (ARVs) immediately, at the same clinic visit. Patients who have any negative results will be referred for further investigation, care, counseling, tests, or other services prior to being dispensed ARVs. Follow-up will be by passive medical record review. The primary outcomes will be ART initiation in  ≤ 7 days and retention in care 8 months after study enrollment.

**Discussion:**

SLATE II improves upon the SLATE I study by reducing the number of reasons for delaying ART initiation and allowing more patients with TB symptoms to start ART on the day of diagnosis. If successful, SLATE II will provide a simple and streamlined approach that can readily be adopted in other settings without investment in additional technology.

**Trial registration:**

ClinicalTrials.gov, NCT03315013. Registered on 19 October 2017.

**Electronic supplementary material:**

The online version of this article (10.1186/s13063-018-2928-5) contains supplementary material, which is available to authorized users.

## Background

In its 2017 revision of the global guidelines for HIV care and treatment, the World Health Organization (WHO) called for rapid or same-day initiation of antiretroviral treatment (ART) for eligible patients testing positive for HIV, with rapid initiation defined as starting treatment within 7 days of diagnosis [1]. South Africa, with the world’s largest national ART program, adopted this recommendation in October 2017 [2]. Neighboring countries such as Zambia [3] and Kenya [4] also allow for same-day initiation in their most recent national guidelines, though they do not recommend it as the default procedure.

The WHO’s justification for its recommendation was to increase the number of patients starting ART, in particular by reducing loss to care between diagnosis and treatment initiation [1]. In many countries, standard procedures for initiation of HIV-infected patients onto treatment remain slow and cumbersome, deterring some patients from starting ART entirely and leading to long delays for others. Anecdotal field reports suggest that there has been some recent improvement in accelerating ART initiation, but multiple pre-initiation visits, long waiting times, stock-outs of supplies, staff absences, and poor communication between staff and patients remain common [5–7].

We previously conducted two studies on same-day treatment initiation in South Africa. The first was the RapIT trial, which relied on point-of-care test instruments to determine patient eligibility under the prevailing CD4 count threshold. RapIT demonstrated that offering same-day initiation could meaningfully increase uptake of ART and viral suppression [8], but the equipment required for RapIT was not feasible for routine care [9]. The second trial, SLATE I, was an evaluation of a clinical algorithm that allowed a clinician to determine eligibility for immediate (same-day) dispensing of antiretroviral (ARV) medications. To be eligible under the SLATE I algorithm, patients could not have any symptoms of tuberculosis (TB)(i.e., any cough, fever, night sweats, or weight loss, or other opportunistic infections); a medical history that indicated previous defaulting from ART, recreational drug use, recent initiation of TB treatment (< 14 days), or specific concurrent medications; physical examination findings that called for additional care; or negative answers to a short readiness screen. SLATE I is being conducted in South Africa and Kenya; the protocol has been described in detail previously [10].

Enrollment for SLATE I was completed in July 2017 in South Africa and April 2018 in Kenya. While follow-up to primary outcomes for that study is still underway, the baseline results indicated a number of potential improvements to the original algorithm. Most important, under SLATE I, exactly half (149/298) of intervention arm patients in South Africa (and 40% in Kenya) were deemed not eligible for same-day initiation for one or more of the reasons mentioned above. Of the 149 South African patients not eligible, nearly three quarters (109, 73%), and 83% of ineligibles in Kenya, had at least one symptom of TB [11], with or without other reasons for screening out. Patients with ≥1 TB symptom were all referred back to standard care for further investigation for TB if regular clinic staff thought it warranted. The study clinics in South Africa diagnosed 8 cases of TB in the intervention arm, resulting in 101 TB-negative patients—roughly a third of the entire intervention arm sample—facing an unnecessary delay in ART initiation. Among the 8 patients diagnosed with TB, all had at least two TB symptoms, and most (7/8) had three or all four symptoms, as well as examination findings indicative of TB. We thus concluded that screening a patient out for mild TB symptoms, without further complications, was too stringent a requirement.

Other aspects of the SLATE I algorithm that were identified as overly restrictive included screening patients out for any self-reported use of recreational drugs, any previous default from ART, or self-reported logistical barriers to maintaining adherence. Each of these criteria ended up screening out patients whom study clinicians believed could safely have been offered same-day initiation.

SLATE I was designed to address the concerns of clinicians and program managers in 2015, when the algorithm was first proposed [12]. In view of both the WHO’s 2017 recommendation and our experience with SLATE I enrollment, we revised the algorithm to incorporate fewer reasons for referring patients back to standard care. We are now beginning implementation of the SLATE II study. In this paper, we describe the protocol for a pragmatic, individually randomized evaluation of the effectiveness of the SLATE II algorithm in increasing ART uptake and retention in care in South Africa, compared to standard care.

The purpose of the SLATE II algorithm is to allow clinics to initiate ART in a streamlined, patient-centered way that minimizes the time required for both patients and staff, reduces loss to follow-up prior to treatment initiation, and increases the proportion of patients who are eligible for same-day initiation. For those who would benefit from additional care or support, the algorithm remains conservative in referring these patients back to standard care, where additional services can be provided.

## Methods/Design

SLATE II is an individually randomized, non-blinded, pragmatic evaluation of the effect of the revised SLATE algorithm on ART initiation and retention in care compared to routine standard care, conducted at primary health clinics in South Africa. Many of the procedures are identical to those in SLATE I, which is described in detail elsewhere [10]. Here we focus mainly on the differences between the SLATE I and SLATE II studies.

### Intervention: the SLATE algorithm II

Like SLATE I, SLATE II tests a clinical algorithm that streamlines and structures the information required from patients before ARVs are dispensed for the first time. The algorithm consists of four “screens”: symptom report, medical history, brief physical examination, and readiness assessment. Patients who “screen in”—have satisfactory responses to all four screens—can then be dispensed ARV medications on the spot, without any further steps or delays. Initiation of ART immediately after completing the four SLATE screens, without any further services required, is labeled “immediate” initiation in this study. Patients who screen out, i.e., have at least one unsatisfactory response on a screen, are referred for further services, such as a laboratory test, more intensive physical examination, or counseling, before ARVs are dispensed.

**Fig. 1:**
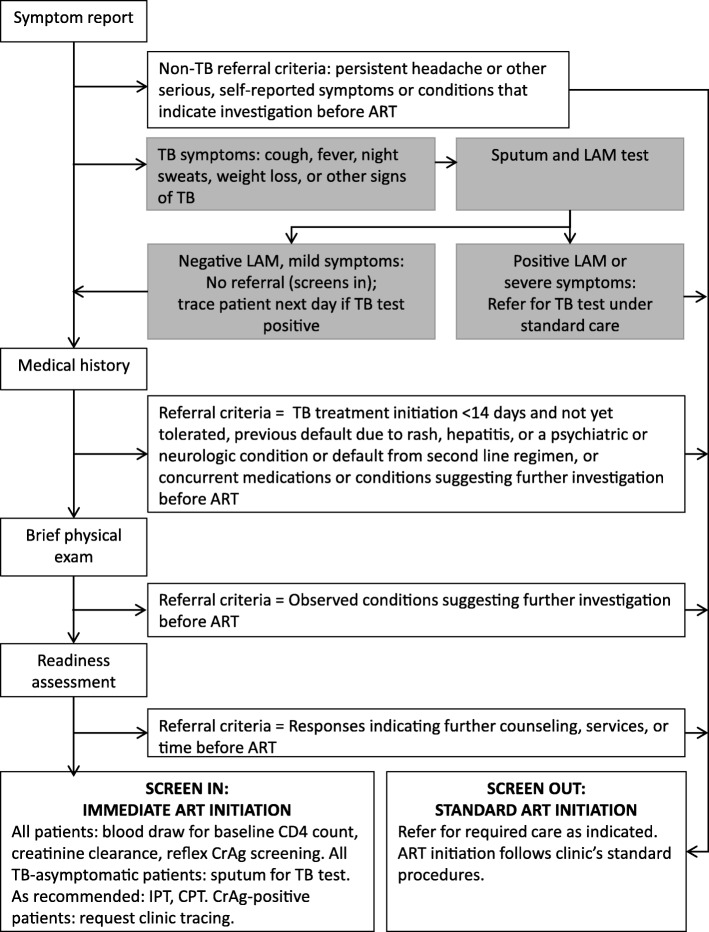
The SLATE II algorithm. *TB* tuberculosis, *LAM* lipoarabinomannan antigen of mycobacteria, *ART* antiretroviral therapy, *CrAg* cryptococcal antigen, *IPT* isoniazid preventive therapy, *CPT* cotrimoxazole preventive therapy

Table 1 and Fig. 1 describe the SLATE II algorithm and screens in detail, with differences between SLATE II and SLATE I shown in the far right column of Table 1. The questions in the SLATE II screening instruments used in the study are available in Additional file 1. Further information about the screens can be found in our previous publication [10].

**Table 1:** SLATE II algorithm screens and differences between SLATE I and SLATE II

Screen	Overall purpose of screen	Reasons for screening out (SLATE II)	Justification	If screen out, anticipated next step	Differences from SLATE I
Symptom report and TB module	Identify self-reported conditions that require additional investigation	Severe TB symptoms regardless of LAM or positive LAM test result	See text for explanation; clinician’s judgment as to seriousness	Referral for TB test	In SLATE I, any TB symptom (cough, fever, night sweats, weight loss) of any duration or seriousness was a criterion for screening out. No LAM test was used. Clinicians in SLATE I were trained to regard any other symptom as a criterion for screening out, rather than only relevant, serious symptoms
		Persistent headache for > 2 days	Symptom of cryptococcal meningitis [31, 32]	Referral for CrAg screening	
		Other serious self-reported symptoms	Other symptoms could indicate the need for further clinical investigation; clinician’s judgment as to seriousness	Referral for additional clinical consultation	
Medical history	Through self-report, identify individuals on concurrent medications or who may struggle with adherence	Started TB treatment within the past 2 weeks and not yet tolerated, based on clinical judgment	Guidelines recommend up to 2 weeks’ delay in ART initiation for patients starting TB treatment to allow them to tolerate the TB medications before starting ART.	ART initiation by the clinic as soon as TB treatment is tolerated	In SLATE I, all TB treatment initiated within 14 days, any prior default from ART, and any report of substance abuse were always criteria for screening out. These were removed and replaced in SLATE II
		Previous ART default due to rash, hepatitis, or a psychiatric or neurologic condition or default from second line regimen	Prescription of a different regimen may be required. Default from first-line ART for other reasons is no longer a criterion for screening out	Referral for additional clinical consultation	
		Concurrent medications or conditions that may create problems for patients	Some medications, ongoing intravenous recreational drug use, or mental illness may interact with ARVs or hamper adherence	Referral for additional clinical or pharmacy consultation	
Physical examination	Record weight, height, temperature and blood pressure and identify any observable serious conditions that require additional investigation	Any conditions that call for further investigation prior to ART initiation	Patient may identify previously unreported symptoms or clinician may observe conditions that indicate a need for further clinical investigation before starting ART; clinician’s judgment as to seriousness	Referral for additional clinical consultation	No major differences. Clinicians in SLATE II are encouraged to use clinical judgment of seriousness of any conditions identified
Readiness assessment	Confirm that the patient feels ready to start ART today	Responses that indicate reluctance, hesitation, or serious concerns in starting and adhering to treatment	Creates a structured opportunity for clinician and patient to discuss any concerns that the patient has not yet raised	Referral for additional counseling and follow-up support as indicated	Logistical reasons for not being ready, such as concern about future transportation to the clinic, are no longer criteria for screening out in SLATE II

*CrAg* Cryptococcal antigen, *LAM* lipoarabinomannan antigen of mycobacteria

### TB screening under SLATE II

The most important difference between the SLATE II and SLATE I algorithms concerns the detection of tuberculosis. As explained above, a large proportion of SLATE I patients who would otherwise have been eligible for immediate ART initiation screened out due to the presence of TB symptoms and the fear that starting ART in patients with active TB would cause unmasking of immune reconstitution inflammatory syndrome (IRIS) [13, 14]. Very few SLATE I patients were found to have TB, however. Upon closer examination of the data and discussion with the study team and local clinicians, and based on new WHO guidelines recommending starting ART immediately in patients with low CD4 counts [1], we concluded that those who screened out due to a passing cough or fever, or due to HIV-related (rather than TB-related) weight loss, were in fact among the patients who would benefit most from immediate ART initiation. Data from SLATE I also suggested that the patients who did have TB had multiple symptoms that were evident in the symptom report and physical examination. Finally, published evidence and the experience of study clinicians suggest that patients with milder TB symptoms who do have TB are also less likely to develop serious complications even if they start ART with undetected TB [15]. We thus decided to take a different approach in SLATE II, in the hope of allowing a much larger proportion of patients to start ART on the same day.

As described in the shaded boxes in Fig. 1, there is now an additional module for patients who report any TB symptom(s) during the symptom report. Symptomatic patients are tested using a lipoarabinomannan antigen of mycobacteria (LAM) test, which is a point-of-care urine test that requires approximately 25 min to administer [16]. LAM has been evaluated in multiple settings [16–20] and is recommended for patients with low CD4 counts [21]. Research in Kenya found that clinical signs plus LAM identified 84% of TB cases in a population of HIV-infected outpatients with CD4 counts below 200 cells/μl [19]. The LAM test has been found to be far more sensitive among very ill patients, typically those who are hospitalized or have CD4 counts < 200 cells/μl, than among healthier patients. These are also the patients who are most likely to have more severe immune reconstitution reactions to ART if it is initiated before or at the same time as TB treatment, as lower CD4 counts are strongly correlated with risk of IRIS. LAM is thus most effective in the population that is most at risk of IRIS. It is also inexpensive—less than US$3 per test [22]—and easy to use at point of care, requiring less than half an hour to run and no capital equipment. LAM is not currently in use for routine TB screening or diagnosis in South Africa, but the National Department of Health of South Africa has indicated that it intends to incorporate LAM into national guidelines as soon as more operational research is available to guide how best to utilize the test [23]. We thus judged it appropriate to include as a component of the SLATE II algorithm.

In addition to having a LAM test, symptomatic patients are also asked in-depth questions about the duration and severity of their symptoms, and concerns are investigated during the physical exam. Any patient with a positive LAM test, TB symptoms that are severe or of long duration, or other clinical indications of TB is screened out and referred back to standard care for further TB testing. Patients who have negative LAM results and milder symptoms are not screened out due to TB but are given an information card about TB that reminds them to return to the clinic right away if their TB symptoms worsen or new symptoms appear.

After completing all four SLATE II screens, all intervention arm patients, symptomatic or not, are asked for a sputum sample for TB testing. In South Africa, samples are sent to a National Health Laboratory Service (NHLS) lab on the day they are provided, and results from Xpert *Mycobacterium* tuberculosis (MTB)/resistance to rifampicin (RIF) testing are available electronically the next day, though it is typically several days before clinics receive and record these results. For SLATE II intervention arm patients, study staff access the electronic NHLS portal the next working morning to check results. Any patient with a positive test is then contacted by study or clinic staff (up to three attempts by phone, followed by a home visit) and asked to return to the clinic to initiate TB treatment as soon as possible.

### Outcomes, randomization, and sample size

SLATE II has two primary outcomes. For primary outcome 1, we will estimate the proportion of patients in each arm (baseline and intervention) who initiate ART within 7 days of study enrollment. SLATE I allowed up to 28 days to achieve this outcome, but current global and South African recommendations both call for initiation within 1 week of a patient’s first clinic visit. A patient who has not initiated within 7 days will be regarded as failing to achieve this primary outcome. Secondary outcomes, as described below, will also allow proportions of patients initiating ART at any interval after study enrollment to be assessed.

For primary outcome 2, we will estimate the proportion of all patients in each arm who initiate ART within one month (28 days) *and* are alive, in care, and retained on ART 8 months after study enrollment. Eight months was selected for SLATE I to allow up to 1 month to initiate ART, 6 months of follow-up after treatment initiation, and up to 1 month to return for the 6 months routine clinic visit. We retained the full 8-month period for SLATE II to allow comparison of results between the two studies.

Although viral suppression is preferable to retention in care as a measure of ART success, we are not confident that all the study sites will consistently perform routine viral load tests at 6 months, and viral suppression will be considered as a secondary outcome. To allow for the irregularity of clinic visits, we will allow any clinic visit between 5 and 7 months after treatment initiation (or between 6 and 8 months after study enrollment, taking into account the month allowed for treatment initiation) to represent the 6 months visit. Other secondary outcomes are described in Table 2. Most are similar to those for SLATE I but are now stratified by TB symptom status.

**Table 2:** Outcomes of the SLATE II study

Outcome	Justification and/or further description	Data analysis
Primary outcomes		
ART initiation within 7 days of study enrollment	WHO calls for initiation within 7 days for all patients	Intention-to-treat analysis; comparison of proportions between groups presented as a risk difference and 95% confidence intervals
Initiated ART within 28 days of study enrollment *and* alive, in care, and retained on ART 8 months after study enrollment	Retention at 8 months captures early attrition on ART, in case the manner of ART initiation affects longer-term outcomes (8 months was selected to allow up to 1 month to initiate ART, 6 months of follow-up after treatment initiation, and up to 1 month to return for the 6 months routine clinic visit)	Intention-to-treat analysis; comparison of proportions between groups presented as a risk difference and 95% confidence intervals. Reasons for not achieving this outcome will also be described to the extent that routinely collected follow-up data allow
Secondary outcomes		
ART outcomes		
ART initiation within 14 days of study enrollment for TB suspects	SLATE II aims to avoid delay of ART initiation in patients with mild TB symptoms	Intention-to-treat analysis; comparison of proportions between groups presented as a risk difference and 95% confidence intervals
ART initiation within 1, 14, and 28 days of study enrollment	Both national and global guidelines recommend same-day initiation (1 day). Since other published studies have used 14 and 28 days, maintaining this secondary outcome will allow comparison with SLATE II results	Intention-to-treat analysis; comparison of proportions between groups presented as a risk difference and 95% confidence intervals
Time to initiation, in days	Time to initiation captures any effect of SLATE II on accelerating initiation	Intention-to-treat analysis; comparison of time to initiation presented as survival curves with log rank test
Viral suppression by 8 months after study enrollment, for all patients and for TB suspects	Allows ≤1 month (28 days) to initiate ART, 6 months of follow-up after treatment initiation, and ≤ 1 month to return for the 6 months routine clinic visit	Intention-totreat analysis; comparison of proportions between groups presented as a risk difference and 95% confidence intervals. Reasons for not achieving this outcome will also be described to the extent that routinely collected follow-up data allow
Retention in care 14 months after study enrollment, for all patients and for TB suspects	Allows ≤1 month (28 days) to initiate ART, 12 months of follow-up after treatment initiation, and ≤ 1 month to return for the 12 months routine clinic visit; any visit 12–14 months after study enrollment will represent the 12 months visit	Intention-to-treat analysis; comparison of proportions between groups presented as a risk difference and 95% confidence intervals
SLATE II algorithm performance		
Proportions of study patients who screen in and screen out for immediate ART initiation using SLATE II algorithm criteria	Will provide guidance on proportions of patients who could be initiated under SLATE II if adopted as routine care	Intention-to-treat analysis; comparison of proportions between groups presented as a risk difference and 95% confidence intervals
Reasons for ineligibility	Will provide guidance on types of referral services required from clinics	Descriptive analysis of proportions of patients screening out for each possible reason indicated on SLATE II screens
Frequency and types of adverse events	Will indicate probability of adverse events related to the algorithm and guidance on what to expect	Descriptive analysis of adverse events reported in medical records after ART initiation for each follow-up period
Patient preferences on the speed and timing of ART initiation	Baseline questionnaire data	Descriptive analysis of medians and interquartile ranges (IQRs) for continuous outcomes and proportions and corresponding 95% confidence intervals for categorical outcomes
TB outcomes		
Proportion of symptomatic patients who test positive for TB using the LAM test	Will determine usefulness of using a point-of-care LAM test as part of ART initiation	Descriptive analysis of proportion of tests found positive
Proportions of symptomatic and asymptomatic patients who test positive for TB using Xpert MTB/RIF	Will determine usefulness of testing asymptomatic patients with Xpert as part of ART initiation	Descriptive analysis of proportion of tests found positive
Health system outcomes		
Costs to patients of ART initiation under standard and intervention procedures	SLATE II is hypothesized to reduce the number of clinic visits required for ART initiation and thus costs to patients	Sum of clinic visit costs and time spent from enrollment visit to visit at which ARVs are dispensed, calculated from questionnaire responses
Costs to providers of ART initiation under standard and intervention procedures and cost-effectiveness of intervention	SLATE II is hypothesized to reduce the number of clinic visits required for ART initiation and thus costs to providers	Estimate of provider costs using previously described [33] bottom-up costing methods, with resource utilization extracted from medical records and case report forms (CRFs) and unit costs obtained from study sites. The average cost to the provider per patient achieving each primary outcome will be compared between intervention and standard initiation groups to provide an estimate of the cost-effectiveness of the two strategies. Costs will be reported as means (standard deviations) and medians (IQRs) in local currencies and US dollars
Comparison of SLATE I and SLATE II results	Assess changes between the original and revised algorithms and to look for secular changes in patient characteristics, clinic procedures, and patient outcomes over time	Direct comparison of findings between the two studies wherever possible (Note: If standard arm results change significantly between SLATE I and SLATE II, may conduct a difference-in-differences analysis, with SLATE I serving as the “pre” period and SLATE II as the “post” period)

Study patients will be randomized 1:1, using block randomization in blocks of six, to the intervention arm or standard care arm. Blinding is not possible in a pragmatic evaluation such as SLATE, as each arm will follow very different procedures post-randomization.

SLATE II is powered on the first primary outcome (initiation within 7 days) but will allow enrollment to increase as needed to achieve the first secondary outcome (initiation within 14 days for TB suspects). Using results of the SLATE I study and conservatively assuming that time to initiation will decrease under standard care in response to the new WHO and South African guidelines mentioned above, we estimate that 60% of treatment-eligible patients will be initiated on ART within 7 days in the standard arm, and we consider an increase to 75% to be programmatically important. Using an α of 0.05, power of 90%, 1:1 randomization, and an uncorrected Fisher’s exact test will require a minimum sample size of 200 patients per arm. We increased our total sample size to 600 to ensure sufficient power if our estimate for the standard group is too low and to allow stratification in the analysis.

### Study sites and ethics review

SLATE II will be conducted in the same three public sector clinics in Gauteng Province of South Africa as was done in SLATE I. All three serve urban informal settlements typical of peri-urban settings. Under our local partner, the Health Economics and Epidemiology Research Office (HE^2^RO) of the University of Witwatersrand, the study will employ at each site a Public Health Nurse; this is the professional cadre that initiates patients onto ART in South Africa. Study nurses will be trained on study procedures but have no additional clinical training or qualifications beyond what is typical in routine clinic settings. Each site will also have two study assistants, who will be responsible for screening for study eligibility, obtaining informed consent, administering the baseline questionnaire, and performing other non-clinical tasks.

The study protocol, which is available as an additional file (Additional file 2), has been approved by the Boston University Institutional Review Board and the University of the Witwatersrand Human Research Ethics Committee (Medical) (Additional file 3). It is registered with ClinicalTrials.gov as NCT03315013.

### Screening and enrollment

At each site, we will recruit approximately 200 adult patients (≥ 18 years) who have tested positive for HIV, either at the current clinic visit or previously, and have not yet initiated ART. Pregnant women will be excluded, as procedures for initiating and managing pregnant women on ART differ from those for non-pregnant adults. Patients who intend to receive further HIV care at a different clinic rather than the study site will also be excluded, as will those who are determined by study staff to be physically, mentally, or emotionally unable to consent. We note that these study eligibility criteria will allow enrollment of patients at varying points in the HIV care cascade. Some will be enrolled a few moments after having their first positive HIV test, while others will have been aware of their HIV status and potentially attending pre-ART monitoring visits for several years. The study sample will thus reflect the full range of ART-eligible patients presenting at South African clinics.

Site staff will refer patients with HIV to the study assistant for study screening and consent. Patients will be screened consecutively in the order in which they are referred. All patients who are found eligible for the study will be asked for written informed consent (Additional file 4) and enrolled in the study. Following consent, female patients will be asked to complete a pregnancy test, and any who are found to be pregnant will be withdrawn from the study and escorted to the site’s antenatal clinic to enroll in antenatal care and prevention of mother-to-child transmission care.

Enrollment in SLATE II is expected to be completed in a 6-month period in 2018. The first primary outcome can then be estimated just 7 days after the last patient has been enrolled, while the second primary outcome will require 8 months of follow-up. Each outcome may then take a month or more to be recorded in the electronic record system. We thus expect most study results to be available by the end of 2019.

### Procedures

**Fig. 2:**
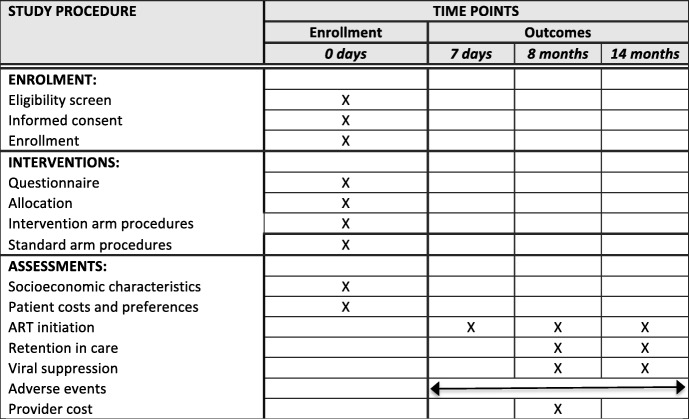
SPIRIT figure

With the exception of the TB module described above, study procedures for SLATE II are identical to those in SLATE I. Procedures are summarized in Fig. 2 and in the Standard Protocol Items: Recommendations for Interventional Trials (SPIRIT) checklist (Additional file 5), and a full description is available in our earlier publication. SLATE II procedures are summarized more briefly here, with an emphasis on differences between the two protocols.

Prior to randomization, participants will be administered a baseline questionnaire, with sections on demographic and socioeconomic status, costs incurred per clinic visit, preferences for the timing and speed of ART initiation, and TB symptoms. By inquiring about TB symptoms prior to randomization, something not done in SLATE I, we will be able to compare rates and outcomes of symptoms between the study arms. We were not able to do this in SLATE I because TB symptom status is not routinely recorded in standard arm patients’ clinic records.

Randomization assignments will be kept in sequential order in sealed envelopes and conveyed to participants by the study assistants after completion of the questionnaire. Patients allocated to the standard arm will receive a payment equivalent in value to US$12 to thank them for participating and compensate them for their time. The study assistant will then escort standard arm patients back to the appropriate location in the clinic to follow the clinic’s regular schedule of procedures and visits for ART initiation. Most standard arm patients will have no further interaction with study staff after this point. The exception will be any who are identified too far back in the study clinic’s queue to have a blood draw completed before the final laboratory sample pickup from the clinic on that day. Because the time required for study enrollment may have pushed these patients farther back in the queue, the SLATE II study nurse will draw a blood sample from any such standard arm patients.

Patients allocated to the intervention arm will be introduced to the study nurse, who will administer the four SLATE screens. TB symptom questions will be asked again, as part of the symptom report. Most patients are expected to complete all four screens in 15–20 min, with the exception of those with TB symptoms, who will be administered the TB module.

Following administration of the four screens, intervention arm patients will have a blood draw for a CD4 count and creatinine clearance test. Blood samples will be sent for processing at the same laboratories used in routine care. Although baseline CD4 counts are no longer used to establish ART eligibility in South Africa, they continue to be performed for all patients initiating ART. Baseline CD4 count remains a strong indicator of early outcomes on ART and is therefore an important variable for the study analysis [24, 25]. At this time, patients in the intervention arm will also be given instructions for producing a sputum sample and asked to provide a sample for TB testing, regardless of symptom status—this was not done in SLATE I.

The results of the four screens will indicate whether a patient has either screened in or screened out of the SLATE II algorithm. Findings in any of the four screens that suggest that further clinical investigation, counseling, laboratory tests, or other services are advisable prior to dispensing ARVs will cause a patient to screen out. Patients who screen out will be referred for the suggested follow-up services and escorted to the appropriate clinic location for follow-up as soon as possible. Unlike SLATE I, the SLATE II algorithm encourages the study nurses to use their own clinical judgment in assessing seriousness of reasons for screening out. If, for example, a patient is a previous defaulter and seems likely to continue to face challenges with adherence, the nurse may opt to initiate ART immediately and refer the patient for additional counseling after starting ART. This contrasts with SLATE I, where previous default would automatically have caused the patient to screen out.

As with SLATE I, screening out of the SLATE II algorithm will not necessarily preclude same-day ART initiation. Some patients who screen out will receive the additional service at the same clinic visit and can still be prescribed ARVs by the clinic before completing the visit. We anticipate that this will happen less frequently in SLATE II, however, as patients who screen out of the SLATE II algorithm are likely to have more serious barriers to ART initiation than did those in SLATE I.

Patients who “screen in”—have satisfactory responses to all four SLATE screens, do not require any additional services, and are eligible for immediate dispensing of ARVs—will have a brief conversation with the study nurse to confirm that the patient remains ready to start ART, understands what happens next in the study, and has no further questions or concerns. The nurse will then write a prescription for an initial supply of medications and dispense the medications directly from the nurse’s room. Patients in the intervention arm will then receive the same token of appreciation as was provided to patients in the standard arm.

### Data collection and management

For the primary analysis, the two main sources of study data will be case report forms (CRFs) and patients’ routine medical records. The SLATE CRF will be programmed in REDCap Mobile (https://www.project-redcap.org/software/mobile-app/) and completed on tablet computers.

All data for the follow-up period, until all primary and secondary outcomes are reached, will come from routinely collected medical records, primarily TIER.Net, South Africa’s national HIV monitoring system [26]. To complete missing fields in TIER.Net, we will also search patients’ paper files and clinic registers as needed. To allow linking of data between the CRF and medical records and tracing of patients with positive lab results, a separate linking form with identifiers will be completed for each patient immediately after consent. Further details about individual and aggregate data collection, quality review, and access are described in our previous publication [10].

Data generated by the study (CRFs) will be made available in de-identified format following closure of the protocol in a publicly available repository, to be identified in papers published from the study. Data obtained from the study sites (routinely generated medical record data) will not be owned by the study and cannot be made publicly available by the authors.

### Data analysis

Our primary analysis will be an intention-to treat comparison of treatment initiation ≤ 7 days and retention at 8 months by study arm. We will first look for baseline differences between treatment arms in characteristics that predict the outcome to ensure baseline comparability. We will then compare the intervention to the standard arm as a simple comparison of proportions and calculate risk differences with corresponding 95% confidence intervals. If we identify any baseline imbalances, we will use adjusted log-binomial and linear regression models to control for any potential confounding.

We will also look for absolute effect measure modification by important potential modifiers. The main modifier of interest for SLATE II is baseline TB symptoms. We will also look for modification by demographic and clinical characteristics (e.g., age, sex, baseline body mass index (BMI), CD4 count, site). Our analysis for effect modification will use a simple stratification of the primary analysis by the potential modifier and report crude risk differences and risk ratios and their corresponding 95% confidence intervals.

Analytic methods for secondary outcomes are described in Table 2.

### Limitations

We anticipate that the SLATE II study will have four main limitations. First, in order to reach the target sample size in the desired timeframe, there will be heterogeneity in the population enrolled, with some patients who have already made several pre-ART visits and others who were diagnosed with HIV on the day of study enrollment. Second, ART-eligible patients who visit the study clinics but are determined by study staff to be too emotionally distraught (e.g., by just having learned of their HIV diagnosis) or physically ill to be asked to participate in a study will be excluded, which may shift the study sample toward patients who are physically or emotionally healthier than the overall population. Third, by drawing blood samples for standard arm patients who would otherwise be too far back in the clinic queue, we are slightly enhancing standard care. We expect this to affect a very small number of patients, however, and the degree of enhancement is minimal. And fourth, as in most operational research studies, we will have little control over what happens in the non-intervention arm. With the advent of the recommendation of same-day initiation in November 2017 and the introduction of “fast track” initiation under the 2015 National Adherence Guidelines [27, 28], there is general momentum to accelerate the initiation process in South Africa. However, enrollment into SLATE II is expected to take only 6 months; therefore, we do not expect major changes in standard care during enrollment.

## Discussion

With global funding for HIV control plateauing [29] but the number of people eligible for antiretroviral therapy continuing to rise [30], finding innovative approaches to improving the efficiency of HIV service delivery is a high priority. One step in the HIV care cascade that still requires optimization is ART initiation. Building and improving on our experience in SLATE I, the SLATE II algorithm aims to maximize the number of patients who qualify for same-day initiation, while still identifying those who require additional care prior to initiation. If successful, SLATE II will provide a simple and streamlined approach that can readily be adopted in other settings without investment in additional technology.

The SLATE II algorithm improves on SLATE I by attempting to distinguish between TB-symptomatic patients who do have TB and those who do not. The existing TB symptom screen is both non-sensitive and non-specific, resulting in delays in ART initiation for a large number of patients who do not have TB and failing to identify a small number of patients who do. SLATE II addresses these limitations with detailed questions and a LAM test for patients with symptoms and by requesting a sputum sample from all patients, regardless of symptoms. To minimize any risk of IRIS created by initiating mildly symptomatic patients on ART before TB test results are available, the study includes both additional information for symptomatic patients and active tracing of anyone with a positive TB test. SLATE II also allows immediate initiation for some categories of patients who automatically screened out under SLATE I but may be among those who can benefit most from same-day initiation, such as previous defaulters and those reporting logistical problems in making clinic visits.

Like SLATE I, SLATE II has the potential to reduce the time and resources that both providers and patients must invest in ART initiation, while also diminishing the likelihood that patients will be lost from care between diagnosis and treatment initiation. If it can do so without jeopardizing outcomes after starting ART, then SLATE II will offer national HIV programs and providers an improved and easily adapted approach to an important component of HIV care.

### Trial status

The trial status details are as follows: Protocol Version 1.0, October 5, 2017; enrollment start date March 14, 2018; enrollment completion date September 18, 2018. ClinicalTrials.gov NCT03315013, registered October 19, 2017,

https://clinicaltrials.gov/ct2/show/NCT03315013.

## Additional files

Additional file 1: SLATE II screens. (PDF 67 kb)

Additional file 2: SLATE II protocol. (PDF 2300 kb)

Additional file 3: Ethics approvals. (PDF 553 kb)

Additional file 4: Informed consent form. (PDF 134 kb)

Additional file 5: SPIRIT checklist. (PDF 507 kb)

## Abbreviations

ART: Antiretroviral treatment
BMI: Body mass index
CrAg: Cryptococcal antigen
LAM: Lipoarabinomannan antigen of mycobacteria
SLATE: Simplified Algorithm for Treatment Eligibility
TB: Tuberculosis
WHO: World Health Organization
