# Supplementary material for: Improved simplified clinical algorithm for identifying patients eligible for immediate initiation of antiretroviral therapy for HIV (SLATE II): protocol for a randomized evaluation
Source: Trials. 2018 Oct 11;19:548. doi: 10.1186/s13063-018-2928-5 (PMC6180640; doi:10.1186/s13063-018-2928-5)
Supplement: Supplementary file 1 — SLATE II screens. (PDF 67 kb) [file 13063_2018_2928_MOESM1_ESM.pdf]

SLATE II CRF Part 2

Study ID ______________________________
(Scan the study ID barcode on the outside of the randomization envelope.)

Study Clinician: "Hello, my name is _______. I will be collecting the rest of the information we need for the study, to see if you can start ART now, as part of the study, or if you should receive additional care or counseling from the clinic first. I will start by asking you if you have any symptoms of illness today."

Only patients randomized to the intervention arm should continue!

REDCap User

Time started ______________________________

SYMPTOM REPORT

Study nurse: please review symptoms reported by patient in CRF Part 1 and confirm that each symptom reported is correct or that patient has no symptoms.

Does patient currently have a cough?
◯ Yes
◯ No
(TB module will open at end of symptom report.)

Does patient have a fever now or in the past 24 hours?
◯ Yes
◯ No
(TB module will open at end of symptom report.)

Does patient report having night sweats?
◯ Yes
◯ No
(TB module will open at end of symptom report.)

Does patient report losing weight?
◯ Yes
◯ No
(TB module will open at end of symptom report.)

Does patient report any other symptoms that may indicate TB?
◯ Yes
◯ No
(TB module will open at end of symptom report.)

Does patient have a continuous headache that hasn't gone away for at least two days?
◯ Yes (screens out of immediate ART initiation)
◯ No
(If headache is persistent or continuous, answer "yes." If occasional or temporary, answer "no.")

Does patient report any other symptoms of illness?
◯ Yes
◯ No

If yes, what other symptoms?

____

If yes, do any of the other symptoms indicate that further consultation is needed before starting ART?
- ◯ Yes
- ◯ No

Comments on symptom report

____
(Describe any symptoms reported by patient (duration, intensity, etc.))

TB MODULE: Patient answered "yes" to one or more of the TB symptoms listed above. The TB module will now open and should be completed before continuing with the symptom report.

If the patient already gave a urine sample for a pregnancy test then the LAM test should be performed using that sample. If no urine sample was collected earlier then do so now.

Has patient been asked for a urine sample for a TB LAM test?
- ◯ Yes
- ◯ No

If no, why not? __________________________

Did patient provide a urine sample for a LAM test?
- ◯ Yes
- ◯ No

If no, why not? __________________________

Was LAM test performed?
- ◯ Yes
- ◯ No

If no, why not? __________________________

LAM test result
- ◯ Negative
- ◯ Positive

Duration of cough
- ◯ 1 day
- ◯ 2 days
- ◯ 3-7 days
- ◯ 8-14 days
- ◯ >14 days

Description of cough
- ◯ Productive
- ◯ Non-productive

Duration of fever
- ◯ 1 day
- ◯ 2 days
- ◯ 3-4 days
- ◯ >4 days

Description of fever

____

Duration of night sweats
- ◯ last night only
- ◯ 2-3 nights
- ◯ 4-7 nights
- ◯ >7 nights

Description of night sweats

____

How much weight have you lost, in kg? ________________________________

For how long have you been losing weight, in weeks? ________________________________

Description of weight loss ________________________________

Description of other symptoms that might indicate TB ________________________________

Do any single symptoms or combination of symptoms suggest that further investigation or a TB test are needed before ART initiation?
- ◯ Yes
- ◯ No

On the basis of symptoms, this patient screens out for immediate ART initiation and will be referred for additional care. Complete remaining 3 screens and the other sections in CRF Part 2.

MEDICAL HISTORY

Have you been on ART before?
- ◯ Yes
- ◯ No

If yes, when did you start taking ART before? ________________________________
(Enter year YYYY)

If yes, when did you stop taking ART before? ________________________________
(Enter month MM and year YYYY)

If yes, why did you stop taking ART before? ________________________________

Based on timing of or reason(s) for default, what do you recommend for this patient?
- ◯ Start ART immediately (today, in the study), no further steps required
- ◯ Start ART immediately (today, in the study), refer for additional counseling or other services
- ◯ Do not start ART immediately (screens out)

(Study nurse completes.)

If you recommended NOT starting ART today due to previous default, explain your decision. ________________________________
(Study nurse completes.)

Are you currently on TB treatment?
- ◯ Yes
- ◯ No

If yes, how many days ago did you start taking TB treatment? ________________________________
(Enter number of days. If more than 1 month, enter number of months with "M")

If you started taking TB treatment less than 14 days ago, have you had any health problems or unpleasant side effects from the TB treatment?
- ◯ Yes
- ◯ No

If yes, describe the problems or side effects. ________________________________

Is the patient tolerating TB treatment well enough to start ART today?
◯ Yes
◯ No
(Study nurse completes.)

Have you been told that you have other diseases or health problems besides HIV?
◯ Yes
◯ No

If yes, what other diseases or conditions do you have?
____

If yes, do any of the diseases or conditions suggest further consultation is needed?
◯ Yes
◯ No

Are you currently taking any anti-epilepsy medication?
◯ Yes (screens out of immediate ART initiation)
◯ No

Are you currently taking warfarin?
◯ Yes (screens out of immediate ART initiation)
◯ No

Are you currently taking any other medications?
◯ Yes
◯ No

If yes, what other medications are you currently taking?
____

If yes, do any of the medications suggest that further consultation is needed?
◯ Yes (screens out of immediate ART initiation)
◯ No
(Study nurse completes.)

On the basis of medical history, should ART be delayed for this patient?
◯ Yes
◯ No
(If treatment should be delayed, complete all four screens, blood draw, payment and completion form and then escort the patient to regular clinic visit.)

Comments on medical history
____

On the basis of medical history, this patient screens out for immediate ART initiation and will be referred for additional care. Complete the 2 screens remaining sections in CRF Part 2.

Physical exam

Temperature
____
(Number entered must have 1 decimal place. Example - '32.0')

Systolic blood pressure
____

Diastolic blood pressure
____

Height (cm)
____
(Number entered must have 1 decimal place. Example - '160.5')

Weight (kg)

____
(Number entered must have 1 decimal place. Example - '60.2')

Did the patient report symptoms that should be examined?

◯ Yes
◯ No

After examination, do any symptoms suggest that further consultation is needed?

◯ Yes
◯ No

If yes, which symptoms should be investigated further?

____

Does the patient have other conditions that further consultation is needed?

◯ Yes
◯ No

If yes, what are the other conditions?

____

On the basis of the physical exam, should ART be delayed for this patient?

◯ Yes
◯ No
(If treatment should be delayed, complete all four screens, blood draw, payment and completion form and then escort the patient to regular clinic visit.)

Comments on physical exam

____

On the basis of the physical exam, this patient screens out for immediate ART initiation and will be referred for additional care. Complete the last screen below and the remaining sections in CRF Part 2.

Readiness assessment

If you should start ART today, how ready are you?

◯ Not ready (screens out of immediate ART initiation)
◯ Thinking about trying, maybe in the next month or so (screens out of immediate ART initiation)
◯ Almost ready, maybe this week (screens out of immediate ART initiation)
◯ Ready today
(If response is not ready or thinking about trying, delay required; if almost ready, discuss with patient and decide; if ready, continue)

How will you remember to take your medication every day?

☐ Mobile phone reminder
☐ Alarm on a clock or watch
☐ Ask someone to remind me
☐ Use a calendar or diary
☐ Take my tablets at the same time as I do something else every day (like brushing my teeth)
☐ No reminders, I'll just remember
☐ Other way (specify below)

If other, specify

____

Is there anything that will stop you from taking your tablets every day?

____

Did the patient raise any issues or concerns that lead you to think that ART should be delayed?

◯ Yes (screens out of immediate ART initiation)
◯ No
(Study nurse completes.)

If yes, what were these issues or concerns and how did you respond?

____
(Study nurse completes. Please write a detailed description)

On the basis of the readiness assessment, should ART be delayed for this patient?

◯ Yes
◯ No
(If treatment should be delayed, complete all four screens, blood draw, payment and completion form and then escort the patient to regular clinic visit.)

Comments on readiness assessment

____

Time ended

____

On the basis of the readiness assessment this patient screens out for immediate ART initiation and will be referred for additional care. Complete the remaining sections in CRF Part 2.

CD4 COUNT

Blood drawn for study CD4 count?

◯ Yes
◯ No - patient already had bloods drawn today
◯ Refused blood draw

Blood sample bar code (1)

____

What labs were bloods drawn for?

☐ CD4
☐ CRE
☐ HBV
☐ FBC
☐ RPR
☐ ALT
(Check all labs that bloods were collected for)

SPUTUM SAMPLE

All patients in the intervention arm should be told how to produce a sputum sample and asked for one.

Was patient able to produce a sputum sample?

◯ Yes
◯ No
◯ Refused to try

Sputum sample barcode

____
