# Supplementary material for: Improved simplified clinical algorithm for identifying patients eligible for immediate initiation of antiretroviral therapy for HIV (SLATE II): protocol for a randomized evaluation
Source: Trials. 2018 Oct 11;19:548. doi: 10.1186/s13063-018-2928-5 (PMC6180640; doi:10.1186/s13063-018-2928-5)
Supplement: Supplementary file 2 — SLATE II protocol. (PDF 2300 kb) [file 13063_2018_2928_MOESM2_ESM.pdf]

SLATE II

Research Protocol

Randomized Evaluation of a Revised, Simplified Clinical Algorithm for Identifying Patients Eligible for Immediate Initiation of Antiretroviral Therapy for HIV (SLATE II—SIMPLIFIED ALGORITHM FOR TREATMENT ELIGIBILITY II)

BUMC IRB Protocol Number: H-37010 (United States)
University of the Witwatersrand Human Research Ethics Committee (Medical) Number: 171011.

Federal-Wide Assurance Numbers:

FWA00000301 (Boston University Medical Campus)
FWA00000715 (University of the Witwatersrand)

October 5, 2017

Version 1.0

Table of Contents

1. Summary **4**
2. Investigators **5**
3. Background and rationale **6**
3a. Background 6
3b. Rationale for SLATE algorithm 7
3c. Rationale for SLATE II study 8
3d. Proposed revisions to the original algorithm 10
4. Objectives **12**
4a. Primary objectives 12
4b. Secondary objectives 13
5. Study Design **13**
5a. Overview 13
5b. Study sites 15
5c. Study population 15
6. Enrollment and Preparation **16**
6a. Identification of potential study patients 16
6b. Study screening 16
6c. Consent 16
6d. Pregnancy test 16
6e. Questionnaire 17
6f. Randomization 17
7. Intervention **18**
7a. Algorithm data collection 18
7b. TB module 19
7c. Blood draw 20
7d. Sputum sample collection 20
7e. Referral of patients who screen out 21
7f. Dispensing of ARVs and prophylaxis 21
7g. Completion of direct study interaction and payment 21
8. Medical Record Follow Up **22**
9. Data Collection and Management **22**
9a. Sources of data 22
9b. Study screening form 23
9d. Case report form 23
9e. Medical record data 25
9f. Study identification numbers and linking of records 26
9g. Data entry and storage 27
10. Staff Supervision and Training **27**
11. Sample Size **28**
12. Outcomes and Data Analysis **28**
12a. Outcomes 28
12b. Analytic methods 29
12c. Dissemination of findings 31
13. Human Subjects Considerations **31**
13a. Risks and protection against risks 31
13b. Benefits 35
13c. Costs and payments to subjects 35
13d. Recruitment 35

13e. Informed consent 36
13f. Protection of confidentiality 36
14. References **36**
12. Appendices **37**

1. Summary

In its 2017 revision of the global guidelines for HIV care and treatment, the World Health Organization called for rapid or same-day initiation of antiretroviral treatment (ART) for eligible patients testing positive for HIV. In sub-Saharan Africa, where most HIV patients are located, studies continue to document high losses of treatment-eligible patients from care before they receive their first dose of antiretroviral medications (ARVs). Among facility-level reasons for these losses are treatment initiation protocols that require multiple clinic visits and long waiting times before a patient who tests positive for HIV is dispensed an initial supply of medications. Simpler, more efficient, accelerated algorithms for ART initiation are needed, including strategies for rapid initiation in patients with symptoms of tuberculosis, most of whom do not have active TB.

In July 2017, the original SLATE study (SLATE I) completed enrollment in South Africa. One of the most striking findings of the study so far is the large proportion of patients who "screened out" of the SLATE algorithm and were referred for additional services rather than started on ART immediately. Among 298 patients assigned to the intervention arm and evaluated for immediate treatment eligibility under the SLATE algorithm, 149 (50%) screened out, two thirds of these (100/149) due to symptoms of TB. The vast majority of the TB suspects (93/100, 93%) tested negative for active TB.

The SLATE II study will revise the original SLATE algorithm to provide a pathway for immediate ART initiation for some patients with TB symptoms. Under SLATE II, patients with TB symptoms will be clinically evaluated by the study nurse and will receive a urine point of care LAM (lipoarabinomannan antigen of mycobacteria) test. Those with milder symptoms and a negative LAM test will be offered immediate ART. Those with more serious symptoms and/or a positive LAM test will be asked to return the next day to receive TB test results and either immediate ART or TB treatment. All intervention arm patients (symptomatic and asymptomatic) will be asked for a sputum sample for Xpert testing, and positives will be contacted on the next day. The SLATE II algorithm will also incorporate other improvements identified from SLATE I.

SLATE II will be a pragmatic, individually randomized evaluation to determine the effectiveness of the revised algorithm in increasing ART initiation, compared to standard care, among non-pregnant adult patients. Six hundred HIV-infected adult patients not yet on ART will be enrolled during a routine clinic visit and randomized to receive the intervention or standard care. Patients in the intervention arm will be administered a revised version of the SLATE screens, including the TB add-on; those found eligible under the algorithm will be offered immediate treatment initiation, while those who are not eligible will be referred for standard clinic care. Patients in the standard arm will be referred for ART initiation under standard clinic procedures. All care after the initial visit will be by the clinic under standard care. Primary outcomes will be ART initiation within 7 days of study enrollment and viral suppression within 8 months of study enrollment.

The study will be conducted at three healthcare facilities (clinics) in South Africa. In September 2017, the South African National Department of Health instructed all clinics to offer same-day ART initiation to eligible patients but provided little guidance on determining eligibility. SLATE II will help to create such guidance. If successful, it will improve on the SLATE approach to collecting and interpreting a minimum set of patient data that will avoid delaying treatment initiation for the majority of patients who are eligible for immediate ART, while deferring initiation in the minority who should not start immediately.

2. Investigators

Sydney Rosen
Research Professor
Boston University School of Public Health
Boston, MA, USA
Role on study: principal investigator

Matthew Fox
Professor
Boston University School of Public Health
Boston, MA, USA
Role on study: co-investigator, statistical advisor

Alana Brennan
Research Scientist
Boston University School of Public Health
Boston, MA, USA
Role on study: co-investigator, study manager

Bruce Larson
Research Professor
Boston University School of Public Health
Boston, MA, USA
Role on study: co-investigator

Francois Venter
Associate Professor
Department of Medicine
University of the Witwatersrand
Johannesburg, South Africa
Role on study: co-investigator, clinical advisor

Mhairi Maskew
Principal Researcher
Health Economics and Epidemiology Research Office
University of the Witwatersrand
Johannesburg, South Africa
Role on study: South Africa principal investigator, data analyst

Lungisile Vezi
Study Coordinator
Health Economics and Epidemiology Research Office
University of the Witwatersrand
Johannesburg, South Africa
Role on study: Study coordinator

3. Background and rationale

3a. Background

In its 2017 revision of the global guidelines for HIV care and treatment, the World Health Organization called for rapid or same-day initiation of antiretroviral treatment (ART) for eligible patients testing positive for HIV[1]. The South African National Department of Health adopted this recommendation in October 2017[2]. Neither organization provided detailed guidance, however, on how to implement the recommendation.

In sub-Saharan Africa, where most HIV patients are located, studies continue to document high losses of treatment-eligible patients from care before they receive their first dose of antiretroviral medications (ARVs). Among facility-level reasons for these losses are treatment initiation protocols that require multiple clinic visits and long waiting times before a patient who tests positive for HIV is dispensed an initial supply of medications. Simpler, more efficient, accelerated algorithms for ART initiation are needed, including strategies for rapid initiation in patients with symptoms of tuberculosis, such as a cough, most of whom do not have active TB.

Although it remains an under-studied topic, attention is gradually turning toward the "how" of rapid and same-day treatment initiation. Several published studies were cited in the new WHO guidelines, including our own RapIT study in South Africa[3] and a similar study in Haiti[4]. Based on experience with RapIT, in 2015 we convened a technical consultation that explored approaches to accelerating treatment initiation and developed the first version of a clinical algorithm to allow immediate initiation without waiting for laboratory test results.[5] A more recent editorial that accompanied the release of the Haiti study also emphasized the importance of developing approaches for implementing same-day initiation.[6]

In July 2017, the original SLATE study (BU H-35634; Wits 160910) completed enrollment in South Africa, with a final sample size of 602. The SLATE algorithm for simplified treatment eligibility is shown in Figure 1. The algorithm aimed to identify patients who should and should not start ART immediately, at the same clinic visit, based on symptoms, medical history, a physical examination, and patient readiness.

Figure 1: Original SLATE algorithm from SLATE I study

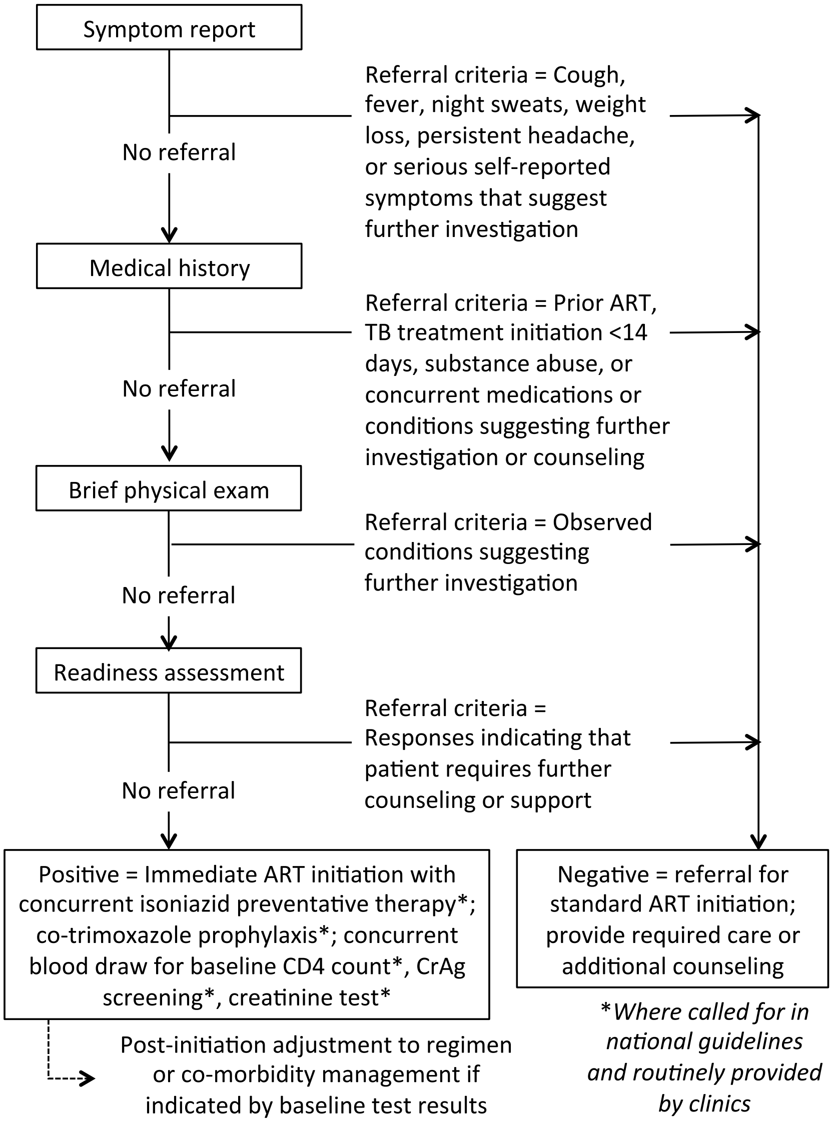

SLATE follow-up for primary outcomes is still underway, but the baseline (enrollment) data provide valuable information. Based on these data, we propose to conduct the follow-on study described in this protocol.

3b. Rationale for SLATE algorithm

The fundamental rationale for developing (and improving upon) a same-day initiation algorithm remains the same as it was for the SLATE I study and is illustrated in Figure 2. In many countries, standard care procedures for initiation of HIV-infected patients onto treatment are slow and cumbersome, requiring multiple clinic visits and long waiting times at each visit. These serve as barriers to treatment initiation, and many patients disappear from care between having an HIV test and initiating ART. The purpose of the SLATE studies is to create a practical, standardized approach to collecting and interpreting a minimum set of patient data that will avoid delaying HIV treatment initiation for the majority of patients who are eligible for immediate ART, while deferring initiation in the minority who should not start

immediately. SLATE II will use baseline data from SLATE I to improve the SLATE algorithm to allow a larger proportion of patients to start ART on the same day as their first clinic visit, without jeopardizing the welfare of those who need or wish for a delay. It will thus provide information to the South African NDOH on how to implement same-day initiation, which was recommended by NDOH in October 2017.

Figure 2. Rationale for SLATE I and SLATE II studies

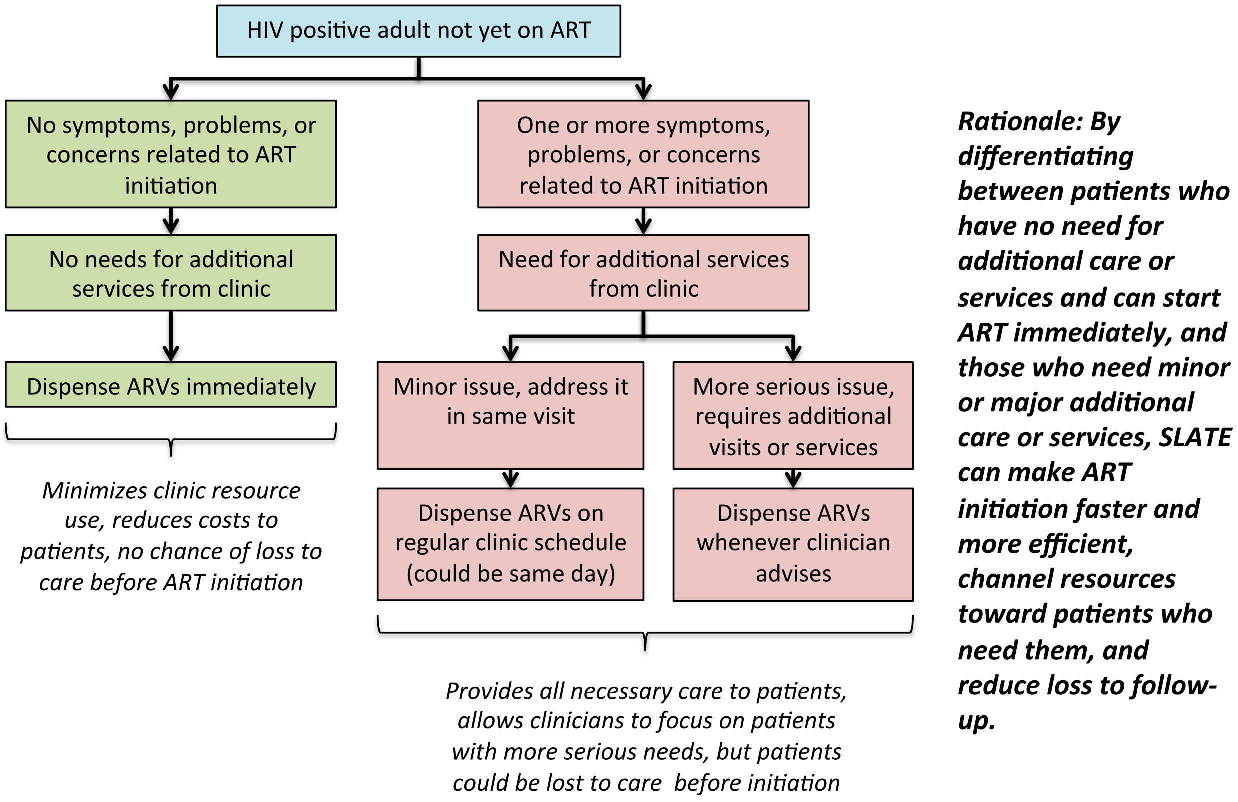

3c. Rationale for SLATE II study

One of the most striking findings from the baseline SLATE I data set is that a larger-than expected proportion of patients "screened out" of the SLATE algorithm—i.e., were referred for additional services rather than started on ART immediately. As Figure 2 illustrates, among 298 patients assigned to the intervention arm and evaluated for immediate treatment eligibility under the SLATE algorithm, 149 (50%) screened out. Just over two thirds of these (100/149) screened out due to symptoms of TB, 11% due to persistent headache, 9% as a result of previously defaulting ART, 4% for substance use, and the rest for other reasons, as shown in the blue box in Figure 2. In Kenya, where SLATE I enrollment is not yet complete, early data suggest an even more pronounced pattern: 46% (27/59) of those in the intervention arm have screened out, and all but one of these (26/27) are due to TB symptoms.

Figure 3: Baseline data from SLATE I study (South Africa)

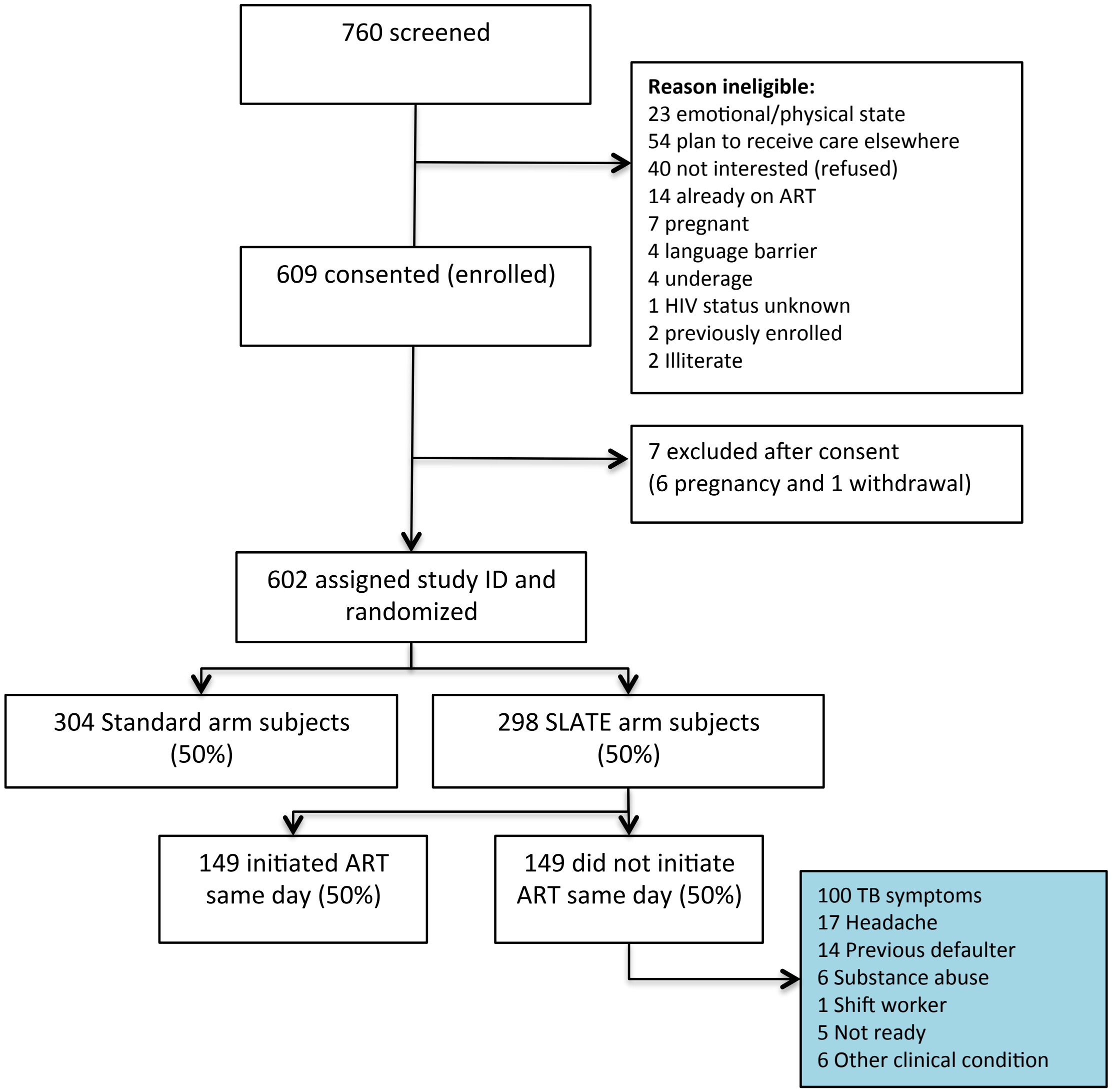

We have traced TB test results for all patients who had TB tests, including all who screened out of the intervention arm due to TB symptoms. Among 100 intervention arm patients with TB symptoms who were screened out and managed under standard care, only 53 completed TB tests (the remainder either were unable to produce a sputum sample, were not offered a TB test by the clinic, or were lost to follow up before having a test). Of the 52 with successful TB tests, 7 (19%) tested TB-positive, meaning that at least 79% and up to 93% of symptomatic patients were screened out unnecessarily. In the standard arm, 102 patients had TB tests, suggesting a nearly identical proportion of symptomatic patients, and only 6 of these (6%) were positive. The overall rate of diagnosed TB among all participants was 2.2% (13/602). The rates of TB positivity among suspects in SLATE I are consistent with or lower than in previously published studies[7,89]. At the same time, other studies have reported finding cases of TB among asymptomatic patients—3.4% in a recent study in Mozambique[8]. The standard symptom screen for TB is neither specific nor sensitive enough to determine whether a delay before ART initiation is required.

There have been a number of recent studies that have examined other approaches to TB screening and testing that may both improve case-finding over the status quo and allow same-day ART initiation among patients with TB symptoms. In Kenya, investigators compared cases identified by clinical signs (symptoms) plus one or more of four tests: chest x-ray, smear microscopy, Xpert MTB/RIF, and a test for lipoarabinomannan antigen of mycobacteria known as LAM, which is a point-of-care urine test that requires approximately 25 minutes to administer[10]. Among these diagnostic methods, only a symptom screen and LAM can readily be performed at point of care while the patient waits; all the others require equipment and expertise that is not available at many primary health clinics and are routinely performed at centralized laboratories, with results available the next day at the earliest. In the Kenya study, the investigators found that clinical signs plus LAM identified 84% of TB cases in a population of HIV-infected outpatients with CD4 counts below 200 cells/μl[11].

The LAM test has been found to be far more sensitive among very ill patients, typically those who are hospitalized or have CD4 counts < 200, than among healthier patients. Both the prevalence and danger of TB are substantially greater among patients with low CD4 counts. These are also the patients who are most likely to have more severe immune reconstitution reactions to ART if it is initiated before or at the same time as TB treatment, as lower CD4 counts are strongly correlated with risk of immune reconstitution. LAM is thus most effective in patients who are most at risk when ART is started in TB suspects, and is thus appropriate for the SLATE II algorithm. In SLATE I, 93/263 (36%) intervention patients with known test results had CD4 counts below 200 cells/μl[11].

In South Africa, the standard of care for TB suspects is to collect a sputum sample and send it for off-site testing using Xpert MTB/RIF, with results available the next day from the testing service. There is often a delay of several days in conveying the results to the patients, and patients who do not return to the clinic may never receive their results. LAM is currently not in use for routine screening or diagnosis, but the Southern African HIV Clinicians Society recommends its use in symptomatic patients with CD4 counts <100[12], and the National Department of Health of South Africa has indicated that it intends to incorporate LAM into national guidelines as soon as more operational research is available to guide how best to utilize the test.[13]

3d. Proposed revisions to the original algorithm

SLATE II will incorporate a revised (improved) screening component for TB, in the hope of increasing the proportion of symptomatic patients who can start ART immediately. As illustrated in Figure 4, all intervention arm patients with TB symptoms will be tested with LAM. Any patients who are LAM-positive and/or have serious TB symptoms, in the study nurse's judgment, will be referred for a TB test without starting ART. Patients who are LAM-negative and have mild TB symptoms will be offered immediate ART initiation. A sputum sample will be collected from all intervention arm patients (with or without symptoms) who are able to produce one and Xpert test results will be collected on the following day. Any patient with a positive Xpert test will be traced on the following day and asked to return to the clinic to initiate TB treatment.

Other aspects of the original algorithm have also been reconsidered in the light of baseline results and the experience of the study team in SLATE I. There is consensus that being a previous ART defaulter from first-line therapy should no longer be a criterion for screening out, as many of the patients who met the definition of defaulter were in fact women who had been placed on ART only for the duration of pregnancy. They did not default care but were taken off it, in compliance with the guidelines prevailing previously. Instead, previous experience on ART will be noted in patients' records with a

recommendation for additional adherence counseling during their first month on ART. Study clinicians will be allowed to screen out and refer for additional care patients whom they believe are at high risk of non-adherence.

There is also agreement that substance abuse, which has included casual recreational users, is not an appropriate criterion for delaying ART initiation in this setting. This criterion will be removed, but study clinicians will be allowed to screen out and refer for additional care patients whom they believe require medical care or other services for their substance abuse. Finally, not all patient-reported barriers to ART adherence will automatically be regarded as criteria for screening out, as they were in the SLATE I study. Study nurses will be asked to use their clinical judgment as to whether a barrier is a valid reason for not initiating ART immediately or is instead something that the clinic and patient can take steps to resolve after initiation.

As shown in Figure 4, the SLATE II algorithm uses the same four screens as SLATE I to assess whether a patient is eligible for immediate (same-day) treatment initiation, but the criteria for screening out are different. Each of the screens is intended to identify specific reasons that a patient might *not* be eligible, including current symptoms of opportunistic infections, previous experiences or behaviors that indicate a need for additional treatment readiness or adherence support, and the patient's own concerns about starting treatment. The algorithm captures all components of treatment eligibility assessment currently used in routine care in South Africa with the exception of pre-initiation laboratory results.

Figure 4. SLATE II algorithm

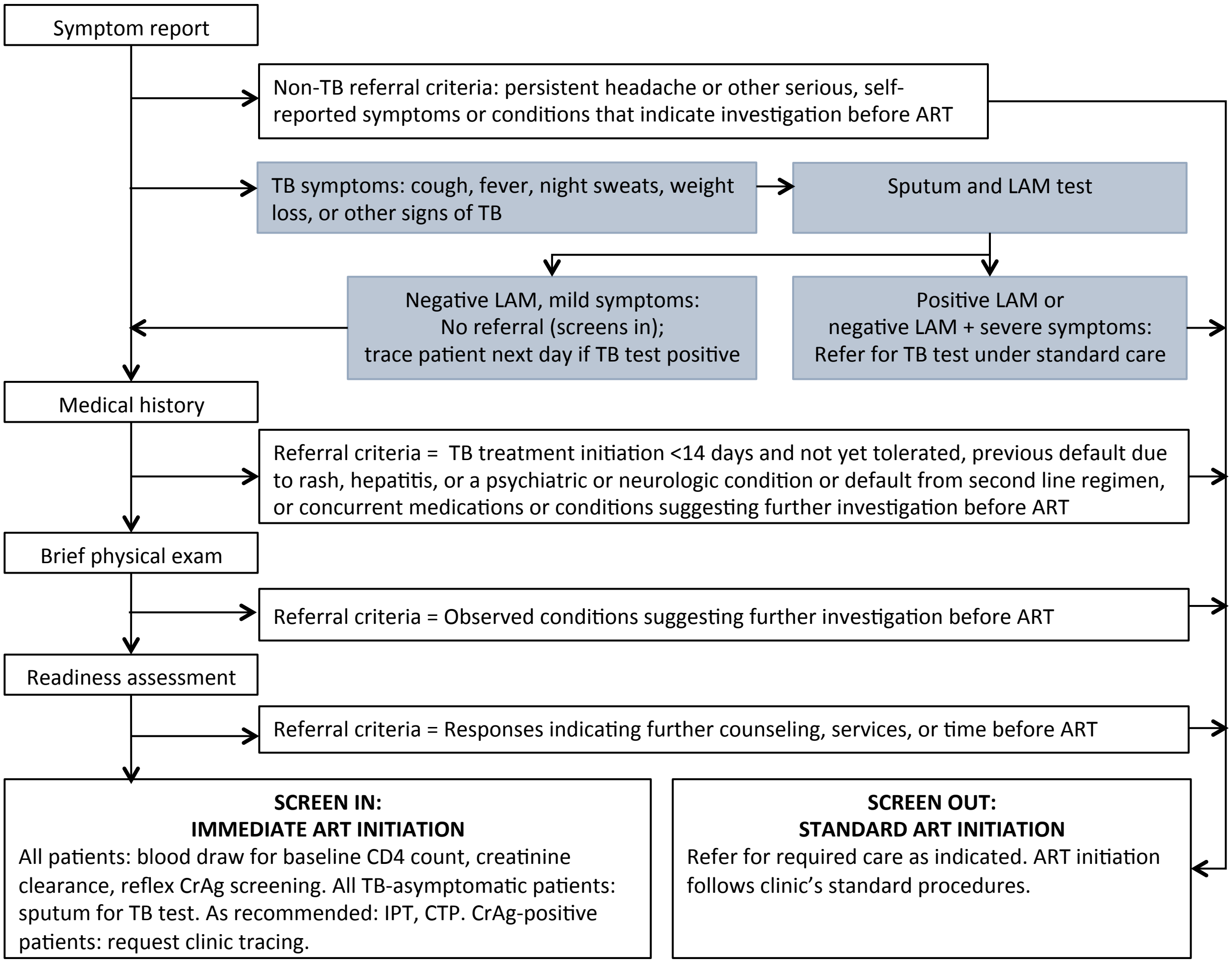

4. Objectives

The SLATE II study is a pragmatic, individually randomized evaluation to determine the effectiveness of the SLATE II algorithm in increasing ART initiation and retention, compared to standard care, among non-pregnant adult patients. Patients will be individually randomized to either an intervention arm, in which the SLATE II algorithm will be implemented, or a standard arm, in which they will receive standard of care treatment initiation.

4a. Primary objectives

The primary objectives of this study are to:

1. Compare the proportion of study-eligible patients who initiate ART within 7 days of study enrollment between HIV-infected patients offered immediate ART initiation under the SLATE algorithm and patients offered standard ART initiation.

2. Compare the proportion of study-eligible patients who initiate ART and are alive, in care, and retained on ART by eight months after study enrollment between HIV-infected patients offered immediate ART initiation under the SLATE algorithm and patients offered standard ART initiation.

4b. Secondary objectives

The secondary objectives of this study are to:

3. Estimate the proportion of study-eligible patients who initiate ART within 1 day (same-day), 14 days, 28 days, and 90 days of study enrollment.
4. Compare the proportion of study-eligible patients who initiate ART and are virally suppressed eight months after having an HIV test or enrolling in HIV care. (Note: viral suppression is a secondary rather than primary outcome because viral load test data are expected to be incomplete.)
5. Compare the proportion of study-eligible patients who initiate ART and are alive, in care, and retained on ART by 14 months after study enrollment between HIV-infected patients offered immediate ART initiation under the SLATE algorithm and patients offered standard ART initiation.
6. Estimate the proportion of HIV-positive patients presenting at study clinics and not yet on ART who are eligible or ineligible for immediate initiation using SLATE algorithm criteria.
7. Describe reasons for ineligibility for immediate initiation, among those found ineligible in the intervention arm.
8. Estimate average time interval in days between study enrollment and ART initiation for each study arm.
9. Describe adverse events after ART initiation as reported in patients' routine medical records and follow-up questions for TB-positive patients.
10. Describe self-reported patient preferences on the speed and timing of ART initiation.
11. Evaluate the usefulness of the LAM test as part of the SLATE II algorithm.
12. Compare between arms the proportion of TB-positive patients who initiate and who complete TB treatment.
13. Estimate the proportion of asymptomatic patients who test positive for TB.
14. Estimate costs to patients of implementing the SLATE II algorithm, compared to standard care.
15. Estimate costs to providers of implementing the SLATE II algorithm, compared to standard care.
16. Compare results of SLATE II study to SLATE I study.

5. Study Design

5a. Overview

The study will be an individually randomized evaluation that will collect data from consented study subjects using questionnaires and review of routine medical records for all patients enrolled; a brief physical examination, venous blood draw where not already done under routine care, sputum sample from patients able to produce one for patients in the intervention arm, and LAM test for patients in the intervention arm with TB symptoms. Patients randomized to the intervention arm will be initiated on ART immediately if they screen in under SLATE II or referred for standard care if they screen out under SLATE II. Health facility information that does not pertain to human subjects (e.g. unit costs) will also be collected. Figure 5 summarizes study design and procedures.

Figure 5. SLATE II study procedures

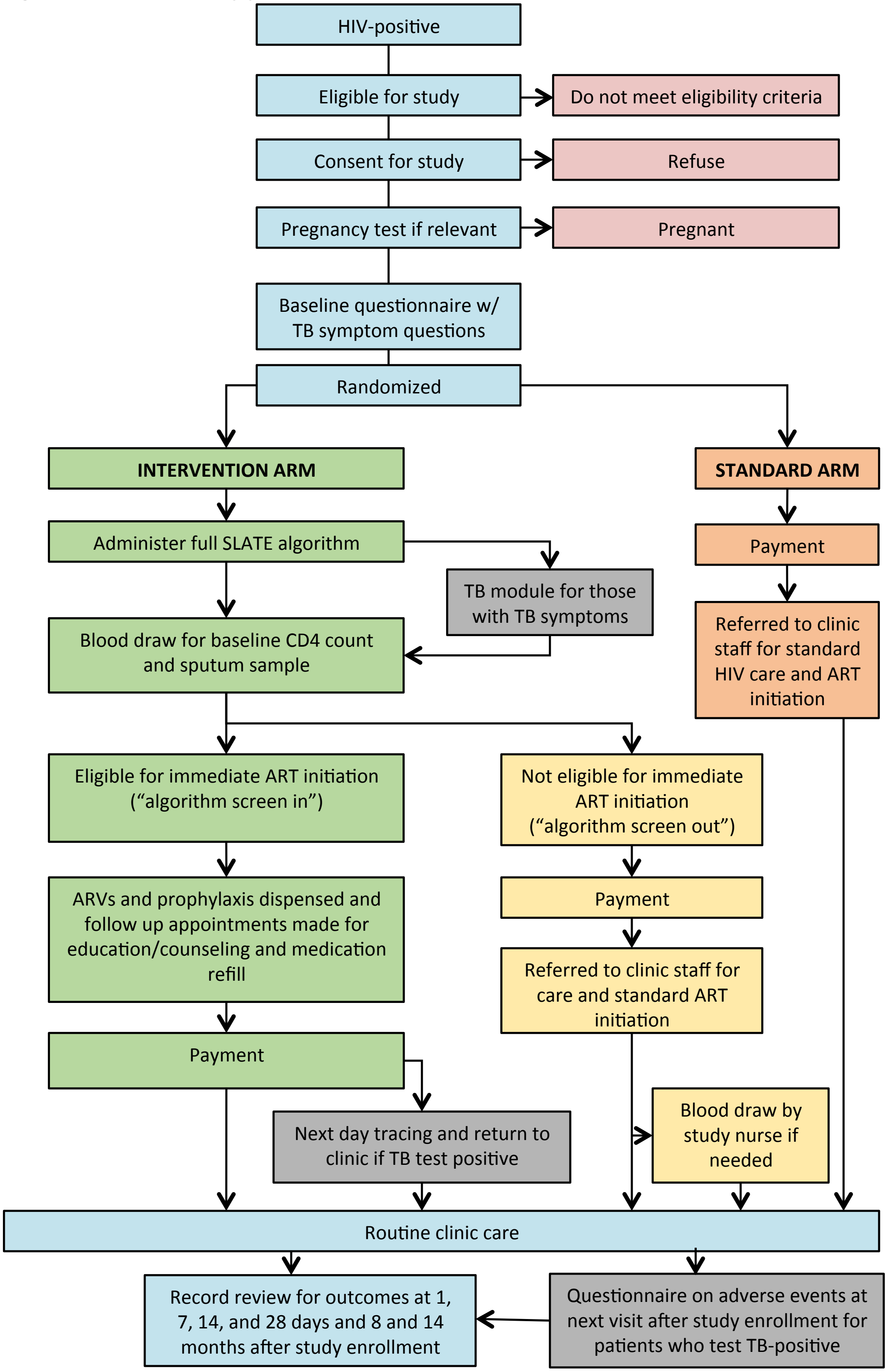

5b. Study sites

The study will be conducted at the same three high-volume, public sector clinics that participated in SLATE I. We selected sites that have both a relatively high volume of potentially study-eligible patients, to reduce the time required for enrollment, and have service delivery infrastructure and staff that are typical of the public sector.

Sites were selected in collaboration with local study teams and in consultation with government health authorities in each area. Formal permission from each site to conduct SLATE II will be submitted to the BU and local IRBs prior to study enrollment at that site.

The study sites are:

- OR Tambo Primary Health Clinic, City of Johannesburg
- Alexandra Community Health Centre, City of Johannesburg
- Jabulani Dumane Community Health Centre, Ekurhuleni Metro

5c. Study population

The study population will be all non-pregnant HIV-infected adults who are not yet on antiretroviral therapy.

Inclusion criteria:
- Adult patients ($\geq$18 years) (initiating children and adolescents on ART is likely to require additional information and adherence support, making the SLATE algorithm less applicable to pediatric populations)
- Confirmed HIV-positive test result at any time (may have been diagnosed previously)
- Self-report that patient is not currently on ART and has not been prescribed ART in the past three months
- Presented at the study clinic for any HIV-related reason or other reason that led to referral for HIV testing or care

Exclusion criteria:
- Pregnant (pregnancy is an exclusion criterion because treatment guidelines for pregnant women differ from those for non-pregnant adults; most pregnant women are diagnosed with HIV and initiated on ART in antenatal clinics, not general adult HIV clinics)
- Not intending to return to this clinic for further HIV care in the coming year (i.e. intends to seek further care somewhere else)
- Not willing to be traced by phone or in person for follow-up care if test results received after the enrollment visit indicate that further care is needed
- Not physically, mentally, or emotionally able to participate in the study, in the opinion of the investigators or study staff
- Not willing or able to provide written informed consent to participate in the study
- Previously enrolled in the same study or the SLATE I study

South Africa has adopted a policy of “treat all” or “test and treat”, so that all HIV-positive individuals are eligible for ART under national guidelines.

Patients will be followed passively through medical record review for up to 14 months after study enrollment.

6. Enrollment and Preparation

6a. Identification of potential study patients

Patients presenting at a study clinic who are HIV-infected but not yet on ART will be eligible for study screening. This includes patients diagnosed earlier and patients newly diagnosed on the same day. At each study site, the relevant clinic staff (counselors, clerks, and/or clinical staff) will inform potentially eligible patients about the study at visit registration or after a positive HIV test. Patients who express willingness to learn more about the study will be referred to a study assistant for screening and consent.

6b. Study screening

Upon referral to the study assistant, patients will receive a more complete description of the study, including the details of why it is being done, study procedures, and the need for written informed consent. They will be assured that participation is voluntary and that they can withdraw from the study at any time, without affecting the quality of care provided by the site. They will also be offered the opportunity to ask questions. If the study assistant concludes that all other inclusion/exclusion criteria have been met, patients will then be asked to provide written informed consent to participate, as described below. The study assistant will complete a screening form (Appendix 1) to confirm study eligibility for each patient screened. The screening form will not collect any identifiable information pertaining to individual patients prior to receipt of written informed consent.

Patients will be screened consecutively, as they arrive at the study site. Due to the volume limitations of study staff, however, recruitment may be halted during intervals when the study assistant and/or study clinician are already busy. It will also be halted in the mid-afternoon where necessary to comply with the site's laboratory pick-up schedule and to ensure adequate time remaining in the day to complete study procedures on the same day. We anticipate that algorithm data collection will take less than 30 minutes per patient, allowing us to enroll and collect data from roughly eight patients per day per site with a single study nurse per site.

6c. Consent

Written informed consent will be obtained from all study participants after study screening and prior to study enrollment. The study information sheet and consent form (Appendix 2) will be translated into Sesotho, isiZulu, and Sepedi, the local languages most commonly spoken by patients at the study sites. Translated consent documents and attestation of consent accuracy will be submitted to the BUMC IRB and Wits HREC prior to use with any study subjects. Potential subjects will be asked if they would prefer to read the information sheet or have it read to them by the study assistant and will be given the opportunity to ask questions about the study. Those who provide written informed consent will be enrolled. Those who refuse consent will be referred back to the clinic staff to complete their routine visit, and the study assistant will indicate the refusal on the screening form.

6d. Pregnancy test

Because SLATE II is not intended for pregnant women, we will conduct a rapid pregnancy test for female patients following consent. Women who have already reported being pregnant will have been screened out prior to consent; the pregnancy test will identify those who were not aware of being pregnant. In the SLATE I study, we identified previously unknown pregnancies among six women, nearly 1% of all patients consented. Patients who have a positive pregnancy test will be withdrawn from the study at this time and escorted to the clinic registration desk to begin antenatal care and PMTCT.

6e. Questionnaire

The study assistant will administer a short questionnaire documenting the subject's basic demographic and socioeconomic characteristics, costs incurred per clinic visit, and preferences regarding ART initiation. The questionnaire will also include the four standard TB symptom screening questions that are used under standard care to identify TB suspects. The presence or absence of TB symptoms cannot be collected from routine medical records, as symptoms are not consistently recorded. Including these questions in the pre-randomization questionnaire will ensure that we have a complete record of TB symptoms in the standard arm, as well as in the intervention arm.

The questionnaire elicits only simple responses and will be completed by the study assistant, not the patient. We therefore do not intend to translate the questionnaire itself into patients' first languages. Instead, for patients who do not speak English, the assistant will ask the questions in one of the commonly-understood languages used by the study population, and then record the answers in English on the questionnaire. The questionnaire is included in the study case report form in Appendix 3.

6f. Randomization

After the questionnaire has been completed, patients will be randomized to the intervention or standard groups of the study. Randomization envelopes will be generated in blocks of 6 and kept at the study sites. For each enrolled patient, the study assistant will open the next envelope in sequential order, read the randomization group, and record it on the case report form. The study assistant will then inform the patient of the randomization assignment.

Following randomization, standard group patients will be escorted to the clinic registration desk to resume their routine clinic visit. The study assistant will repeat the explanation of next steps in the study, which are limited to review of medical records by the study team, provide study payment as described below, and thank them for their willingness to participate in the study. The study team will have no further personal contact with most patients in the standard group. Standard arm patients will receive a hard copy of their TB symptom screen results to give to the clinic staff.

In SLATE I, it was found that standard arm patients occasionally joined the clinic's blood draw queue too late in the day to receive service and were asked to return the next day. Blood samples are not drawn after the last laboratory sample pickup of the day, which is typically at 2 pm. To prevent standard arm patients from being disadvantaged by the study, the study assistant will locate standard arm patients still in the clinic's queue at 1:30 pm and the study nurse will perform a blood draw for them, so that they are not forced to return solely for this purpose. While this could affect study outcomes by encouraging standard arm patients to remain in care, it prevents any cost to patients that could be caused by the time spent enrolling in the study.

7. Intervention

The study assistant will introduce intervention group patients to the study nurse for algorithm data collection. The study nurse will be a qualified public health nurse with the same qualifications as the nurses responsible for ART initiation under standard care at the study sites.

7a. Algorithm data collection

The SLATE II algorithm (illustrated in Figure 4 above) calls for four types of screens—a symptom report, medical history, brief physical exam, and readiness assessment—and a TB module. Each of these is described below and further detail is provided in the case report form in Appendix 3. A negative response to a screen indicates that the patient "screens out" and is referred for additional care (consultation, laboratory test, counseling, other); if all responses are positive, then the patient "screens" in for immediate dispensing of ARVs under SLATE.

1. Screen 1: Symptom report. Prior to randomization, each patient was screened for current cough, fever, night sweats, and weight loss, which comprise the standard, WHO-recommended TB symptom screen[14]. During the symptom report, the study nurse will ask about these symptoms again, screen for persistent (continuous) headache for more two days, which is a symptom of cryptococcal meningitis[15,16], and ask about any other symptoms that the patient has noticed. Patients with persistent headache or other symptoms that suggest further services are needed before starting ART will screen out and be referred to standard care. Patients with TB symptoms will be routed to the TB module. All others will screen in.

2. Screen 2: Medical history: To identify individuals who are likely to face problems with medication-taking or adherence, patients will be asked if they have ever been on ART previously, started TB treatment in the past two weeks, or are taking concurrent medications for epilepsy or warfarin, which can interact with ARVs. Patients who are previous ART defaulters will be asked for the primary reason for stopping treatment earlier. Those who report rash, hepatitis, or a psychological or neurological condition that developed after starting ART, are who defaulters from second-line therapy (rather than first-line), or whom the study nurses judges to be at high risk of defaulting again will screen out, while other previous defaulters will screen in. Patients who have initiated TB treatment in the past two weeks and are judged to be stable and tolerating TB therapy will screen in; those who are not will screen out.

3. Physical exam: A brief, symptom-guided physical exam to look for causes of symptoms or any other evident conditions that could postpone ART initiation will be conducted, and those without such symptoms or conditions will screen in.

4. Readiness assessment and counseling: A published review of readiness instruments for HIV initiation found that none was notably successful in predicting patient readiness for ART, as indicated by adherence once on treatment[17]. Despite this, there is general consensus that explicit attention to readiness is an important component of treatment initiation. Drawing on the published literature on stage-of-readiness scales[18], for SLATE I we created a short instrument which we expected to serve primarily as an invitation to patients to raise concerns or questions about treatment readiness with the clinician conducting the assessment. Of 298 patients in the intervention arm in SLATE I, 5 (<2%) indicated that they were not ready for ART initiation. A revised version of the readiness assessment will be used in SLATE II. Following the questions, the study nurse will have a brief conversation with

the patient to confirm that the patient feels ready to start treatment immediately, understands what happens next, and has no further questions or concerns.

Study nurses will be asked to apply their clinical judgment as they administer all of the screens, to avoid unnecessarily delaying those who are eligible for immediate initiation and to ensure additional care for those who need it. For example, any evidence of recreational alcohol or drug use was a criterion for screening out under SLATE I. In SLATE II, this criterion has been removed, as most of those who screened out were casual, asymptomatic users of non-intravenous drugs. Study nurses will still be able to screen out a patient whom they believe is currently under the influence of alcohol or drugs or is seriously ill or in need of clinic assistance before starting ART, no matter what the cause. Nurses' judgments will be recorded in the study CRF.

If SLATE II were used in routine care, we expect that the screens would be administered sequentially, so that a negative response on the first screen would cause a patient to screen out without having to complete the second screen, and so on. Patients would progress to the next screen only after being found positive on the previous one. For purposes of this study, however, we will administer all four screens, to obtain a complete algorithm data set for each patient in the intervention arm.

Based on SLATE I, we anticipate that for the vast majority of patients, each screen will take less than 5 minutes to administer. Patients who have TB symptoms and/or screen out will require more time, and we therefore estimate needing an average of up to 30 minutes of nurse time per patient in the intervention arm.

7b. TB module

As explained above, 100 patients screened out of SLATE I due to symptoms of tuberculosis, but only 7 cases of TB were found. The new TB module in the SLATE II algorithm aims to reduce the number of patients who screen out due to TB symptoms.

Intervention arm patients who report TB symptoms on the symptom screen will complete the TB module before continuing with the rest of the algorithm. They will first be asked for a urine sample (similar to that required for the pregnancy test) and an Alere Determine™ TB LAM Ag lateral flow strip (LAM) test (http://www.alere.com/en/home/product-details/determine-tb-lam.html) will be performed. For women who have already had a pregnancy test, the same urine sample will be used for LAM. The nurse will then collect additional information about the symptoms reported, such as duration and productivity of cough, timeline of weight loss, etc. TB symptoms will also be prioritized for the physical exam (screen 3), to help identify patients with active TB. The nurse will continue with the next SLATE II screen (medical history) while awaiting the results of the LAM test.

Patients with TB symptoms will screen out of the algorithm if they have a positive LAM test, symptoms or conditions that indicate a high risk of active TB, or both. Nurses will be asked to use clinical judgment to identify LAM-negative patients who should be considered to have TB. Within the study, patients will move on to screen 2 after completing the TB module.

A sputum sample will be requested from all intervention arm patients after the SLATE algorithm has been completed, as described below. The sample will be sent to the laboratory for Xpert testing, with results expected the next day.

7c. Blood draw

Cryptococcal meningitis and other co-infections are more likely in patients with very low CD4 counts. Although South Africa's guidelines do not require the results of a CD4 count to establish treatment eligibility, it continues to use CD4 counts to assess patients' baseline condition. To avoid asking intervention group patients to wait in the clinic's queue for a blood draw, the study clinician will do a standard venous blood draw, following the same procedures that the clinic would otherwise use. Blood samples will be tested by the laboratories that serve the clinics, following routine procedures, and results will be placed in routine medical records so that the study clinics do not have to repeat the CD4 count for the same patients. The clinic may also use the blood sample for other baseline tests recommended in national guidelines, such as creatinine, hemoglobin, and for patients with CD4 counts under 100 cells/mm^3, cryptococcal antigen (CrAg). The clinic will follow routine procedures for laboratory tests; the study will only be responsible for drawing the required blood sample.

Under national guidelines, any patient who has a CD4 count under 100 cells/mm^3 and a positive cryptococcal antigen (CrAg) screening result should begin presumptive treatment for cryptococcal meningitis. Results of the CD4 count and CrAg screening test will only be available several days after study enrollment, however. To facilitate proper follow up of any intervention arm patient who does have a positive CrAg screening test, study staff will closely monitor CD4 count results for intervention arm patients as soon as the results are available from the NHLS. The study team will alert the clinic to any patient who has a positive CrAg screening test. We will also trace these patients immediately to alert them of their status and ask them to return to the clinic for further assessment by clinic staff. As with TB, tracing will be done through calls to patients' mobile phones on the day results are available, followed by a home visit on the second day if the patient cannot be found by phone.

7d. Sputum sample collection

National guidelines in South Africa currently call for a sputum sample to be requested from all patients with TB symptoms, for testing at a central laboratory with Xpert MTB/RIF. Results are typically available the next day from the National Health Laboratory Service's on-line portal, though it may take several days for results to be recorded in patient files at clinics. Patients are usually asked to return for TB test results in a week's time. Some clinics actively trace (call or visit) TB-positive patients who do not return for their results; others do not have the resources to do so.

Under SLATE II, we will request a sputum sample from all patients in the intervention arm, regardless of symptoms. This will allow us to identify TB cases among asymptomatic patients, of whom 3.4% had TB in a recent study in Mozambique[8]. While this is not currently the guideline recommendation in South Africa, implementing it in SLATE II will provide evidence either for or against testing all HIV-positive patients and will thus be a valuable contribution by the study.

Sputum samples will be sent to the National Health Laboratory Service (NHLS) lab the day they are collected and results for nearly all should be available from the NHLS the next morning. SLATE II study assistants will review results for intervention arm patients as soon as the results are posted. If any patients in the intervention arm who were started on ART the previous have TB, they will be traced immediately to alert them of their status and ask them to return to the clinic to start TB treatment. Tracing will be done through calls to patients' mobile phones on the first day, followed by a home visit on the second day if the patient cannot be found by phone.

Patients who did initiate ART on the same day under SLATE II and are found to have active TB will be brought to the clinic's attention for TB treatment. The SLATE I study team clinicians agreed that few if any patients who screened in under the algorithm—and are thus relatively healthy, based on the SLATE screens—will have ARVs discontinued as a result of a TB diagnosis. These patients will instead be initiated onto TB treatment concurrently, and monitored for drug interactions and adherence as needed.

7e. Referral of patients who screen out

After completing all four of the SLATE screens and the TB module where indicated, the study nurse will fill out a brief referral letter that reports any substantive findings, such as a positive LAM test or a physical exam observation. Intervention arm patients who screen out (have negative responses on any of the SLATE screens) will be referred to routine clinic care to address the issue(s) identified and initiate ART under standard care. The study clinician will discuss and explain the concern(s) with the patient and then escort the patient to the registration desk to continue with a standard clinic visit. The patient will be given the referral letter and encouraged to give the letter to the clinic nurse or medical officer. Patients who do not wish study findings to be shared with clinic staff will not be required to pass on the letter, with the exception of LAM test results.

Patients who are referred to routine care due to a negative SLATE screen will remain in the intervention arm, have the same study follow up as other intervention arm participants, and be analyzed as part of the intervention arm.

For patients who screen in (have positive responses on all the SLATE screens and are eligible for immediate ART initiation) and agree, referral letters will be placed in their clinic files along with other source documentation, such as the regular ART record that must be filled out for all new ART patients. Letters will not be filed for patients who do not agree.

7f. Dispensing of ARVs and prophylaxis

Patients who have a full positive SLATE screen (no reasons for delay) will immediately be offered their first supply of ARV medications. While making the offer to dispense, the study clinician will confirm that the patient still wishes to start ART immediately, answer any questions the patient may have, and reiterate the importance of returning for the patient's next appointment and medication refill.

In addition to ARVs, national guidelines call for prescribing isoniazid preventive therapy (IPT) and/or co-trimoxazole prophylaxis to new HIV patients. The study clinician will also write prescriptions for these medications. Where possible, the study clinician will have a stock of medications in the study room, so that the patient does not need to go anywhere else in the clinic to obtain their medications. If this is not allowed, the study clinician or study assistant will escort the patient to the clinic pharmacy to obtain the medications without waiting in a queue. The clinician or study assistant will then escort the patient to the clinic registration desk to schedule the next appointment, which will include participation in one of the clinic's routine adherence education sessions.

7g. Completion of direct study interaction and payment

Direct study interaction with study subjects will cease as follows:

- *Intervention group:* Upon completion of the ART initiation or screen-out steps described above, after which subjects will be managed by the study site following standard procedures. The exception to this is patients who screen in and are then found to have active TB or are CrAg-positive; these patients will be traced through phone calls and home visits to encourage them to return to the clinic immediately to start appropriate treatment.

- *Standard group:* After randomization, at which point subjects will be managed by the study site following standard procedures.

- *All patients with TB*: In both study arms, patients who are found to be TB-positive will be asked to complete a short follow-up questionnaire at their next routine clinic visit between 2 and 6 weeks after study enrollment. The study assistant will keep a record of the next scheduled visit for each patient and will seek the patient in the TB clinic on the scheduled day. The questionnaire (Appendix 5) will focus on any adverse events that have occurred since study enrollment.

From these points on, all subjects, regardless of which type of initiation they received, will be managed by the site following its routine procedures for pre-ART and ART care. Because this study evaluates routine practice and uses retention in care as an outcome, any further study interaction with subjects would affect the results, in particular loss to follow-up. There will therefore be no post-baseline study visits, only routine care visits not involving study personnel. Standard procedures will also be implemented by the study site (not the study team) for study subjects who miss scheduled visits.

The last step in study interaction for both groups will be payment. Reimbursement to enrolled study subjects of R150 (about $11.50) will be provided as a token of appreciation for study participation in the form of a voucher (gift card) to a nearby supermarket and general goods store in South Africa.

8. Medical Record Follow Up

Study subjects in both arms will be followed passively through review of routine medical records kept by the study sites for up to 14 months after study enrollment. This will allow up to 1 month for treatment initiation, up to 12 months of follow up, and up to 3 months for a 12-month routine visit to be made.

The purpose of medical record follow up is to determine which patients enrolled in the study did go on to initiate ART and after what time interval, their early outcomes on treatment, and resource utilization (cost) of initiating ART.

Specific data fields to be collected from medical records are listed under Data Collection below.

9. Data Collection and Management

9a. Sources of data

The study will collect data from four sources, as follows:

1. Screening form. Screening forms will be completed to confirm study eligibility and consent.

2. Case report form (CRF). The study assistant and study clinician will use the CRF to record all information gathered directly from the patient, including the baseline questionnaire, four SLATE screens, TB follow-up questionnaire, and outcomes.

3. Medical records. Routinely collected medical record data will be abstracted from medical records and registers maintained by the study site and by central repositories (e.g. laboratory database) in electronic and paper format.

4. Facility-level records. We will use aggregate information from the study sites to estimate the overall proportion of patients presenting at each clinic who could start ART immediately if SLATE II were used in routine care and the resources that would be required.

9b. Study screening form

After providing a brief description of the study and assuring the patient that participating in screening is voluntary, as indicated in Appendix 1, the study assistant will complete a study screening form for each patient referred by clinic staff. The screening form will record whether the patient meets each inclusion and exclusion criterion and provides written informed consent. For patients enrolled, the Study ID number and medical record number will be added to the screening form, which will become part of the case report form for study subjects. For patients who screen out of the study, it will record the reason for ineligibility but will not contain any identifiers.

For female patients, the results of the pregnancy test conducted after consent will be recorded on the screening form, as pregnancy is an exclusion criterion for the study. No case report form will be opened for patients who have positive pregnancy tests.

9d. Case report form

The case report form will be divided into four parts. The ID form (Part 0) will be collected for all enrolled patients. CRF Part 1 will be collected for all enrolled patients. CRF Part 2 will be collected only for patients randomized to the intervention arm. CRF Part 3, which will be completed after the study visit, will include information on tracing of TB- and CrAg-positive patients; the TB follow up questionnaire, which is only for patients with positive TB tests; and HIV initiation and treatment outcomes for all patients in the study.

<u>ID Form (CRF Part 0)</u>

The study assistant will complete an identification form to help link patient records from disparate sources (e.g. paper file at clinic and electronic record) together and to allow tracing of the patient if a TB test or CrAg test result is positive. The ID form includes:

- Study ID number
- Name
- Date of birth
- Clinic ID number(s)
- National identification number
- Mobile phone number(s) (primary and alternate)

- Physical address(es) (primary and alternative, closest landmark)

CRF Part 1

Part 1, which will be administered by the study assistant, will include:

1. Study ID number.

2. A short, closed-ended questionnaire will be administered by the study assistant after consent. The questionnaire will elicit basic information about:

    - Patient characteristics (age, sex, marital status, household composition, duration living in current location, education)
    - HIV-related information (familiarity with clinic, time of diagnosis, acquaintance with others with HIV or on ART)
    - Activities and employment
    - Costs to patient of making a clinic visit (transport costs, loss of income, etc.)
    - Patient's preferences regarding ART initiation (single visit, multiple visits, intervals between).
    - TB symptoms (cough, fever, night sweats, weight loss).

3. The randomization assignment, which will be added to the CRF Part 1 when the randomization envelope is opened, as described above.

4. A wrap-up form for standard arm patients, to confirm that all remaining steps have been taken for patients allocated to the standard arm.

CRF Part 2

Part 2, the SLATE II algorithm, will be completed by the study nurse for intervention arm patients only. It will include:

1. Study ID number.

2. Symptom report. The symptom report form will record yes/no to the presence of the symptoms listed above and provide a space for the study clinician to describe or comment on symptoms and to state whether the patient is ineligible for immediate ART initiation due to symptoms.

3. TB module. As shown in Figure 5, patients with TB symptoms will receive a LAM test and further investigation of symptoms, the results of which will be recorded in the CRF.

4. Medical history. The medical history form will record answers to the medical history questions and provide a space for the study clinician to describe or comment on these answers and to state whether the patient is ineligible for immediate ART initiation due to medical history.

5. Physical exam. The physical exam form will record findings of the physical exam and provide a space for the study clinician to describe or comment on these findings and to state whether the patient is ineligible for immediate ART initiation due to the outcome of the medical exam.

6. Readiness assessment. The readiness assessment form will record answers to the readiness questions and provide a space for the study clinician to describe or comment on these answers and to state whether the patient is ineligible for immediate ART initiation due to readiness.

7. Blood draw. The blood draw form will confirm that a blood sample was taken and record the identification number or bar code used to identify the sample on the CD4 count laboratory order form.

8. Sputum sample. The study nurse will request a sputum sample from all intervention arm patients. The nurse or study assistant will explain how to do this using visual materials. We anticipate that not all patients will be able to produce a sample.

9. Wrap-up form for intervention arm patients to confirm that all remaining steps have been taken for patients allocated to the standard arm.

CRF Part 3

Part 3 will capture any data for individual patients that is generated after the study visit and is not reliably reported in routine clinic records. Specifically, it will include:

1. Tracing attempts and results for patients with positive TB tests.

2. TB follow-up questionnaire for patients with positive TB tests. Questions will focus on adverse events and medical care obtained since ART initiation.

3. HIV treatment initiation and outcomes. Summary data on key outcomes and dates will be captured to ensure a complete analytic data set despite diverse source data locations.

9e. Medical record data

Once the CRF has been completed, all remaining patient-level data for the study will be collected from routinely maintained medical records. The format of these records may be paper or electronic. All the study clinics enter ART patient data into Tier.net, the national medical record system for HIV. Much of the information we will need, however, will be recorded only in patients' paper records or in clinic registers, and some laboratory results will be recorded only in the NHLS national database. We will access all available records for each study patient, regardless of format or storage location, to try to obtain as complete a study data set as possible. The fields we will aim to capture are listed below.

To be used to link electronic and paper-based records to study identification number only:
- Electronic medical record system number
- Clinic file number(s)
- National ID number
- Name
- Date of birth
- Sex
- Mobile phone number(s)

All subjects at clinic visit on date of study enrollment:

- Date of positive HIV test (if available)
- CD4 count (most recent)
- Date and result of TB symptom screen, if previously done
- Date, type, and result of TB test(s) (current and previous, if any)
- TB treatment, if offered (date of initiation, completion)
- WHO stage and clinical conditions

Subjects who are eligible for ART initiation at the study clinic:

- Reasons for delaying ART initiation, if relevant
- Date ART initiated (first dose of drugs dispensed)
- First-line regimen prescribed

For all clinic visits for duration of study follow up, from study enrollment to the follow-up endpoints indicated above:

- Date of visit
- Primary reason for visit (e.g. pre-ART visit, scheduled medical visit, medication pickup, unscheduled medical visit)
- Types of professionals seen (e.g. nurse, doctor, pharmacy assistant, counselor)
- Group and individual sessions attended (e.g. wellness course, adherence education)
- Numbers and types of all lab tests conducted (including CD4 counts, viral loads, TB test, CrAg test, blood tests to determine ARV regimen)
- Other procedures (e.g. x-rays) performed
- Medications prescribed and dispensed (ARV and non-ARV, including isoniazid and cotrimoxazole)
- Admissions for inpatient care since previous visit, if recorded
- Details of inpatient care received (facility, number of days, medications, lab tests, etc.) if recorded

At follow-up endpoints (6 and 12 months after ART initiation or comparable time point for those who do not initiate ART):

- Date and result of most recent viral load test
- Status at clinic (in care / died / transferred to another site / stopped care / lost to follow up, defined as > 1 month late for last medication pickup)
- New or recurring HIV-related conditions
- Date and cause of death if applicable
- Date and result of most recent CD4 count

9f. Study identification numbers and linking of records

Upon consenting to participate in the study, each subject will be assigned a random study identification number. This will be a five-digit number in which the first digit indicates the site and the remaining four digits are a sequential number that does not provide any identifying information.

A password-protected, encrypted linking file will be created in REDCap to link study identification numbers to subjects' names, dates of birth, national identity numbers, and clinic file numbers. These fields are needed to ensure that study ID numbers can be correctly matched to the identification numbers used in the sites medical records. When a subject has been matched to a clinic record, the

clinic record number will also be added to the linking file. The linking file will be a separate document from any other study files. Access to the linking file will be limited to the study team, and the file will be stored in a secure location separate from the study database.

9g. Data entry and storage

Screening and CRF data will be entered by study staff onto Android-based tablet computers programmed for data collection using the REDCap mobile app. The REDCap system provides a secure, web-based platform for data entry that allows real time monitoring, querying, and quality control of data by an off-site data manager. It allows access to data to be assigned and restricted to individual study staff as needed and creates audit trails to monitor appropriate access. The mobile app allows data to be entered offline and then uploaded to a central server when an internet connection is available. Records are encrypted upon entry and can be removed from data capturers' tablets once they have been uploaded to the server, reducing the chances of breach of confidentiality if a tablet is lost or stolen. REDCap is supported by the Boston University IT office (https://redcap.bumc.bu.edu/) and the Wits University Health Sciences Research Office (https://www.wits.ac.za/health/research/research/staff-and-postgraduate-research-and-statistical-support/).

Medical record data for study subjects will be downloaded from electronic medical record systems where available. The lead data analyst will be responsible for ensuring that study identification numbers are correctly matched to the record numbers in the database. In instances where the data analyst cannot match a study ID number to an electronic medical record number using only the fields in the study linking file, a query will be sent to the on-site study staff, who will access the clinic's paper-based patient files to determine whether the subject ever initiated ART and, if so, locate the correct medical record number.

To assist site and study staff in locating study subjects' files for data extraction, a note or tab indicating that a patient is a participant in the study will be attached to the cover of each subject's clinic file. At regular intervals, files will be pulled and the data fields listed above entered into the study database by study staff. All data entry will take place at the study clinics or other locations where the files are stored; no hard copies of patient records will be taken off site by the study team.

Data will be retained in secure study databases until completion of the study, including completion of all data analysis and report writing associated with the study and closing of the IRB-approved protocol. Study data sets and other electronic files containing study data will then have all identifiers removed and where possible will be made available for use of other researchers through a repository such as Dryad. Hard copies of study documents will be shredded.

10. Staff Supervision and Training

In South Africa, the local study team will include a principal investigator, study coordinator, and site-based study nurses and study assistants. The local PI will be responsible for training and supervising her teams, under the guidance of the overall study PI and other co-investigators. The study nurses who will implement the intervention will have the qualifications required for ART initiation in each study country. Study assistants who will administer consent and the questionnaire will also be trained as HIV counselors to ensure that they are sensitive to the needs of HIV-positive study subjects. We anticipate that most or all of the SLATE II site staff will remain the same as for SLATE I.

All members of the study team will be trained in and adhere to the standards laid out in the Belmont Report and NIH guidelines for research and in research ethics applicable to South Africa. Human subjects ethics training will be repeated on an annual basis to ensure that the study team's understanding of research ethics is current.

11. Sample Size

We will power the study on the first primary outcome (initiation within 7 days) but will allow enrollment to increase as needed to achieve the first secondary outcome (initiation within 14 days for TB suspects).

Using results of the SLATE I study and conservatively assuming that time to initiation will decrease under standard care in response to the new WHO and South African guidelines, we estimate that 60% of treatment-eligible patients will be initiated on antiretroviral therapy on ART within 7 days in the standard group, and we consider an increase to 75% to be programmatically important. Using an α of 0.05, power of 90%, 1:1 randomization, and an uncorrected Fisher's exact test, this requires a minimum sample size of 200 patients per group. Guidelines for standard care have only very recently changed since SLATE I and there are no data on ART uptake under the new guidelines. We will also enroll some patients who will be found ineligible after consent or will withdraw after consent. For these two reasons, we will increase our maximum to sample to 300 per group, or 600 in total, to ensure sufficient power if our estimate for the standard group is too low.

The major difference between the SLATE I and SLATE II studies is the TB module. It is therefore important to be able to analyze initiation for the TB symptomatic subpopulation separately. Secondary outcome 1 is the proportion of suspects who initiate within 14 days. In SLATE I, exactly one third of intervention arm participants had TB symptoms. Among standard arm patients who had TB tests, 62% of patients started ART within 14 days. We consider an increase to 84% (12% absolute difference) to be a reasonable goal for SLATE II. With the same assumptions as above, but limited to TB suspects, we will need to enroll at least 81 TB suspects per arm, which we will adjust to 100 per arm, or 200 in total. This is exactly one third of the overall enrollment of 600. If we reach the maximum of 600 without having enrolled at least 100 TB suspects per arm, we will amend the protocol to allow additional enrollment until the TB suspect target is reached.

12. Outcomes and Data Analysis

12a. Outcomes

The primary outcomes for the study are:

1. The proportion of patients in each arm initiated on ART within 7 days of study enrollment. South African guidelines currently recommend ART initiation within two weeks of a patient's first clinic visit or HIV diagnosis, for those who cannot start on the same day, but international (WHO) guidelines suggest 7 days, and one week is likely to become an international standard. We will estimate the proportion who initiate within 14 days as a secondary outcome.

2. The proportion of patients in each arm who initiate ART and are alive, in care, and retained on ART 8 months after study enrollment. Eight months was selected to allow up to 1 month (28 days) to

initiate ART, 6 months of follow up after treatment initiation, and up to 1 month to return for the six-month routine clinic visit.

Secondary outcomes will include comparisons by arm of:

1. The proportion of TB suspects initiated on ART within 14 days of study enrollment.
2. The proportion of all patients initiated on ART within 1, 14, and 28 days of study enrollment.
3. The proportions of all patients and of TB suspects who initiate ART and are virally suppressed eight months after having an HIV test or enrolling in HIV care.
4. The proportions of all patients and of TB suspects who initiate ART and are alive, in care, and retained on ART 14 months after having an HIV test or enrolling in HIV care.
5. The proportions of HIV-positive patients presenting at study clinics and not yet on ART who are eligible and ineligible for immediate initiation using SLATE algorithm criteria.
6. Reasons for ineligibility for immediate initiation, among those found ineligible in the intervention arm.
7. Median time to ART initiation (days) for each arm.
8. Frequency and types of adverse events reported in medical records after ART initiation for each follow up period
9. Patient preferences on the speed and timing of ART initiation.
10. Proportion of symptomatic patients who test positive for TB using the LAM test.
11. Proportions of symptomatic and asymptomatic patients who test positive for TB using Xpert MTB/RIF.
12. Costs to patients of ART initiation under standard and intervention procedures.
13. Costs to providers of ART initiation under standard and intervention procedures.
14. Comparison of results of SLATE II study to SLATE I study.

12b. Analytic methods

We will first conduct descriptive analyses to allow us to determine the characteristics of the sample we have enrolled. We will then analyze the specific outcomes as described below. For all outcomes, the analytic approach will be by intention-to-treat: subjects will be analyzed according to the intervention they were supposed to receive, whether or not they adhere to the defined intervention. This includes patients randomized to the intervention arm who are screened out of immediate ART initiation by the SLATE II algorithm; they will remain in the intervention arm for data analysis.

Primary outcomes 1 and 2 and secondary outcomes 1-4:

The analysis will begin with a simple comparison of the two treatment groups with respect to baseline predictors of outcomes to look for any imbalances. These potential confounders include demographic (e.g. age, sex) and socioeconomic (education, distance from clinic, employment, etc.) variables, medications, baseline CD4 counts, and low BMI, among others.

We will then conduct a crude analysis comparing the proportion of patients achieving each dichotomous outcome by group and estimating crude risk ratios and crude risk differences and their corresponding 95% confidence intervals. This will be our primary analysis as we anticipate that randomization of over 1000 subjects should lead to balance in baseline covariates. Should any important imbalances occur by chance, we will proceed with an adjusted model as described below for the country-specific analyses.

Next, we will look for effect modification (i.e. subgroup analyses) by important predictors of each outcome, such as age, sex, baseline BMI, CD4 count, and any other important demographic and clinical predictors of outcomes identified. Our analysis for effect modification will use a simple stratification of the primary analysis by the potential modifier and report crude risk differences and risk ratios and their corresponding 95% confidence intervals.

When stratified by these potential effect modifiers, because the sample sizes within groups will be smaller, it is possible that some baseline covariates may be imbalanced by treatment arm. To adjust for potential differences by baseline covariates, a log-linear regression model will be used to estimate adjusted risk ratios. Variables considered to be important in the univariate stage will be included in multivariate models. Adjusted analyses will include covariates which are unevenly distributed across treatment groups and which could plausibly affect retention or ART initiation. Each of these models can then be used to look for patient-level predictors of treatment uptake and retention in care.

Analysis for secondary outcomes 1-4 will be repeated for the subsample of patients with one or more symptoms of TB at enrollment, to examine whether the revised (SLATE II) increases HIV treatment uptake and/or affects treatment outcomes in this critical subpopulation.

Secondary outcomes 5-11:

Secondary outcomes 5-11 are descriptive in nature. These will be described and compared between arms using simple proportions, frequencies, and medians.

Secondary outcome 12:

We will estimate the average cost to patients per clinic visit using questionnaire responses, including transport, substitute labor, and other costs. This cost will then be multiplied by the average number of clinic visits observed in each study arm to generate a total average cost to patients of ART initiation under each initiation strategy. Costs will be reported as means (standard deviations) and medians (IQRs) in local currencies and dollars.

Secondary outcome 13:

At 8 months after study enrollment, all resource usage will be extracted from subjects' medical records and case report forms. Unit costs will be obtained from external suppliers and the site's finance and procurement records and applied to the resource usage data to provide a cost per study patient. Costs will be measured from the provider perspective and will include the cost of all resources utilized for each study patient, including drugs, laboratory tests, outpatient visits, and fixed costs such as building space, equipment, and management staff. We will estimate the average cost to the provider per patient achieving each primary outcome. The average cost per outcome will then be compared between intervention and standard initiation groups to provide an estimate of the cost-effectiveness of the two strategies. Costs will be reported as means (standard deviations) and medians (IQRs) in local currencies and dollars.

Secondary outcome 14:

Most of the primary and secondary outcomes for SLATE II were also estimated in SLATE I. We will compare findings between the two studies wherever possible, both to assess changes between the

original and revised algorithms and to look for secular changes in patient characteristics, clinic procedures, and patient outcomes over time. If standard arm results change significantly between SLATE I and SLATE II, we will also consider conducting a difference-in-differences analysis, with SLATE I serving as the “pre” period and SLATE II as the “post” period.

12c. Dissemination of findings

The results of this study will be disseminated as widely as possible in the study countries, where new strategies for increasing the efficiency of treatment initiation are activity sought. A full report will be made to the study sites and other local stakeholders and will be circulated widely and posted on our website. We will also develop a short briefing document to send to the national and provincial departments of health, donor agencies, and other interested organizations, and we will present the results at relevant conferences domestically and internationally. One or more journal manuscripts will be submitted to an appropriate peer-reviewed international journal.

13. Human Subjects Considerations

13a. Risks and protection against risks

For this study, we will not collect any biomedical samples that are not collected for some or all eligible patients as part of routine care, nor will we implement any clinical procedures that are not routinely carried out under standard of care. The strategy being evaluated primarily reorganizes data collection and accelerates the timing of events. The only non-routine data generated for the study itself will be responses to questionnaires. Study staff will conduct a brief physical exam, collect urine samples, and do a blood draw and sputum collection for intervention arm subjects, but all are procedures that are routinely performed and carry little risk. We therefore believe that our study poses few physical risks to subjects beyond those routinely encountered. There is a small risk of immune reconstitution inflammatory syndrome (IRIS) from initiating on ART patients with active TB, which we address below, and the possibility of risks associated with loss to HIV care, emotional distress, and breach of confidentiality.

TB IRIS

Risk: Starting patients with active TB patients on ART, before treating for TB, can cause immune reconstitution inflammatory syndrome. IRIS may present as a new cough, fever, night sweats, or weight loss or a worsening of these symptoms if they already exist. The risk of IRIS is correlated with low CD4 counts and is thus more common in very sick patients. The risk of IRIS is correlated with low CD4 counts and is thus more common in very sick patients. Even in very sick patients, it is rarely fatal. SLATE II intervention arm patients who screen in under the protocol will not be very sick, as all are ambulatory and any serious symptoms of illness are criteria for screening out and being referred for care before ART initiation. We thus believe that the risk of a serious IRIS reaction is very low. In contrast, we do know that delaying ART in those with low CD4 counts (<50) for more than two weeks is unsafe[19], though there is no guidance on when to start within that two-week interval. We also know from the SLATE I study, as reported earlier in this protocol, that only 2% of patients who meet study inclusion criteria are found to have TB, making this risk applicable to very few participants.

Protection: As is explained above, the TB module introduced in the SLATE II algorithm aims to identify patients at highest risk of active TB, without screening out those with mild TB symptoms who are TB-negative. Protection against the risk of TB IRIS are included at three stages in the SLATE II study. First, as current South African guidelines recommend, we will screen all intervention arm patients for symptoms of TB. Those who have symptoms will receive a LAM test and further physical examination. All patients, with or without symptoms, will be asked for sputum sample for Xpert MTB/RIF testing, which goes beyond current national guidelines. Using patient self-report, empirical examination, the LAM test, and Xpert, we expect to be able to screen out and refer for additional care the vast majority of TB-positive participants.

Second, study staff will monitor laboratory testing results on a daily basis. As explained above, the National Laboratory Health Service usually makes Xpert results available on its protected web portal the day after the sample is submitted. This will allow study staff to check the results of patients enrolled the previous day when they arrive at the study sites in the morning. Any patient who was initiated on ART the previous day and had a positive TB test will immediately be traced by the study staff, informed of the test result, and asked to return to the clinic immediately for further assessment. Clinic staff will also be alerted to expect such patients and to make further tracing attempts if needed. As tracing is currently performed in standard care inconsistently if at all, the study will go beyond current practice to ensure that intervention arm patients are notified of a TB diagnosis.

Third, patients with mild TB symptoms who screen in under the algorithm and are offered ART will be given an information card that reminds them to return to the clinic immediately if their TB symptoms worsen or they develop new symptoms, as could happen with IRIS (Appendix 6). Patients in both arms who test positive for TB will be interviewed at their next clinic visit so that any health problems encountered after starting ART are documented.

Emotional distress

Risk: By necessity, the study population for this study will include some patients who have been newly diagnosed with HIV. Interacting with them in order to explain the study and confirm eligibility before requesting written informed consent may cause some emotional distress for some potential subjects.

Protection: To minimize any emotional distress caused by interacting with study staff or enrolling in the study, we will train the site's staff to emphasize when they introduce the study to potential subjects that referral to study staff and enrolling in the study are completely voluntary and that those who do not wish to enroll will receive exactly the same care as the study site would otherwise have provided. Potential subjects will also be told that they can discontinue participation at any time, even after consenting. Study staff will be trained to look for signs of emotional distress and instructed to terminate or postpone the enrollment process if subjects appear distraught.

Loss to HIV care

Risk: The study aims to initiate on ART patients who might otherwise be lost to HIV care. Some patients lost before or after ART initiation will seek care at other sites, but many will not and

will postpone or end all HIV care and treatment, some until they become more seriously ill, others forever. Although the SLATE II strategy is expected to increase the proportion of patients who do initiate ART, there is a possibility that patients who accept immediate ART initiation under SLATE II will subsequently discontinue treatment at a greater rate than do those who receive standard ART initiation. In our prior study of same-day initiation (RapIT), we found that a higher proportion of patients in the intervention arm than in the standard arm were lost to care, but we believe that most of these were patients who otherwise would never have started treatment at all, had only standard initiation been offered. We therefore expect the SLATE II intervention to have a net positive impact on our primary outcomes. We cannot be certain of this result in advance, however.

Protection: Because this is an evaluation of routine practice and retention in care is a study outcome, no efforts will be made to retain patients in the study, beyond those already routinely undertaken by the study site clinics as part of standard care. Intervention arm patients will be encouraged to remain in the clinic on the day of study enrollment if a routine ART adherence class or club is available on that day. Study staff will escort intervention arm patients to clinic registration desks to confirm that each patient has made an appointment for a follow-up visit and that the first follow-up visit includes the clinic's regular ART adherence activities (e.g. individual counseling, group education sessions, etc.). Through these efforts, intervention arm patients will have access to the same adherence support as standard arm patients, though the support will be offered after ART initiation rather than before.

Loss of confidentiality

Risk: Because we must collect identifying information in order to link CRF data with medical records, accidental disclosure of HIV status or other loss of confidentiality is also possible. A high level of stigmatization continues to inhibit the disclosure of HIV status in the study populations. Data collected in during the study visit or from clinic records could be disclosed and reveal a person's HIV status.

Protection: Several steps will be taken to protect subjects against the risk of accidental disclosure of HIV status. Individual documents or electronic files (including signed consent forms, case report forms, and the linking file) which could associate patients with an HIV study will be kept strictly confidential. Signed consent forms will be stored in locked cabinets away from the study site, with access limited to the senior investigators. CRFs will be held in electronic versions only and will be password-protected. The linking file will also be password-protected, with access limited to study staff. It will be used only for the purpose of linking study identification numbers with clinic medical records.

To protect against other violations of confidentiality, study staff will be trained in expectations that they are not to disclose any information collected in the study to anyone outside the study team. All study staff will be required to pass an ethics certification course, such as the on-line certification offered by the NIH. All study participants will be encouraged to contact the study clinician or clinic manager to report any undesirable conduct associated with the study. These reports will be brought to the attention of the local and international PI, and appropriate steps will be taken to solve the problems, including reporting to relevant ethical review boards.

13b. Benefits

Direct benefits

Study subjects offered immediate treatment initiation will benefit from the opportunity to start ART quickly and with fewer clinic visits and time delays than they would otherwise have incurred. Study subjects offered standard initiation will not receive any direct benefits from the study but will receive the same care that would have been provided in the absence of the study.

Indirect (societal) benefits

The study has the potential to generate substantial indirect benefits to the subjects. The research we will undertake is expected to reveal whether a simplified clinical algorithm can successfully be incorporated into procedures for ART initiation, leading to more patients initiating ART and a reduction in patient attrition from care. The results of the study may thus lead to improvements in HIV care and keep patients alive and in care longer.

Ratio of benefits to risks

The knowledge to be generated by this study will allow HIV treatment programs to determine whether a simplified clinical algorithm can be used to determine eligibility of immediate ART initiation. This study will address an important gap in the current knowledge around ART initiation and what can be done to reduce the already high proportion of patients who are being lost from care after testing positive. There is currently almost no information available on how to reduce these pre-ART losses. As explained in the previous section, we believe that the risk to subjects in our study is minimal and is outweighed by the indirect benefits and the potential importance of the findings.

13c. Costs and payments to subjects

There will be no costs to subjects for participating in this study.

Subjects who consent to participate and complete study procedures will receive a payment of R150 ($11) to reimburse for their time and inconvenience in the form of a shopping voucher for Shoprite/Checkers stores.

13d. Recruitment

Identification of potential subjects will occur during patients' regular clinic visits for any HIV-related service other than monitoring of patients already on ART. At reception and relevant waiting areas at each study site a flier will be posted that informs patients that a study is underway and indicates where to get further information. The flier will be translated into the commonly used languages at the study sites.

The site's counselors, nurses, and registration staff and the study assistants will inform potentially eligible patients about the study while they are awaiting and/or receiving services. They will explain that a study is underway and that patients found eligible are invited to participate. Patients who indicate that they may be interested in participating in the study will be referred to the study assistant for screening.

13e. Informed consent

Written, informed consent will be sought from all study subjects by a trained study assistant. The informed consent information sheet will describe the nature, goals, and procedures of the study and assure subjects their information will be kept confidential. The consent form will explain:

- That patients will not get to decide which group they will be in, but rather that we will decide by chance and they will have a 50-50 chance of being in either group;
- How study procedures will be different from standard care procedures for those randomized to the intervention group;
- How we will do follow-up through medical record data collection; and
- That any individual patient can choose not to participate in the study and be offered standard initiation.

The full informed consent information sheet and form will be translated into Sesotho, isiZulu, and Sepedi, which are the most commonly used languages at the study sites. Translated forms and attestations of translation accuracy will be submitted to the IRB prior to any use of the forms. Subjects who unable to read and/or sign the consent form due to illiteracy will be asked to provide a thumbprint mark, in the presence of a witness who will also sign the form. Patients who do not speak English or any of the languages into which the form has been translated will be regarded as unable to provide written informed consent, which is an exclusion criterion for the study, and will not be eligible for study enrollment. The consent form is attached as Appendix 2.

13f. Protection of confidentiality

Subject data will be captured electronically on case report forms programmed on Android tablet computers using REDCap. The tablets will be password-protected and files will be encrypted. When not in use, they will be stored in locked cabinets at the study sites or in study team offices. Study subjects will be assigned sequential study ID numbers upon consent, and data files will contain study ID numbers only, without any other individual identifiers.

A password-protected linking file allowing medical record data to be matched to study-generated data will be maintained in a secure location, with access limited to study staff. Medical record data will be downloaded or extracted from paper records on a regular basis, as needed for data analysis. Electronic medical record datasets may include data on all patients at the study clinic, not just those included in the study, as it may not be possible to select only study participants prior to downloading. As soon as datasets are downloaded, the linking file will be used to select study participants and link their records with their study identification numbers. All identifiers will then be stripped from the clinical data and records for all patients not enrolled in the study will be deleted.

14. References

1 World Health Organization. GUIDELINES FOR MANAGING ADVANCED HIV DISEASE AND RAPID INITIATION OF ANTIRETROVIRAL THERAPY. Geneva: WHO, 2017.

2 Pillay Y. Same-day antiretroviral therapy (ART) initiation for HIV positive patients. 2017.

3 Rosen S, Maskew M, Fox MP, *et al.* Initiating antiretroviral therapy for HIV at a patient's first clinic visit: the RapIT randomized controlled trial. *PLoS Med* 2016; 13: e1002015.

4 Koenig SP, Dorvil N, De JG, *et al.* Same-day HIV testing with initiation of antiretroviral therapy

versus standard care for persons living with HIV : A randomized unblinded trial. *Plos* 2017; 14: e1002357.

5 Rosen S, Fox MP, Larson B, *et al.* Accelerating the uptake and timing of antiretroviral therapy initiation in sub-Saharan Africa: an operations research agenda. *PLoS Med* 2016; 13: e1002106.

6 Geng EH, Havlir D V. The science of rapid start — From the when to the how of antiretroviral initiation. *PLoS Med* 2017; 14: e1002358.

7 LaCourse SM, Cranmer LM, Matemo D, *et al.* Tuberculosis Case Finding in HIV-Infected Pregnant Women in Kenya Reveals Poor Performance of Symptom Screening and Rapid Diagnostic Tests. *J Acquir Immune Defic Syndr* 2016; 71: 219–27.

8 Ciccacci F, et al. TB diagnosis in HIV+ patients in Mozambique. A field study of combined point-of-care evaluation with GeneXpert, LAM and symptom screening (TUPEB0370). *IAS 2017* 2017; : 370.

9 Selibas K, Hanrahan CF, Deery C, *et al.* TB suspects with negative initial Xpert. *3rd SA TB Conf* 2012; 12–15 June.

10 Mathabire S, Cossa L, Mpunga J, Manhica I, Amoros Quiles I, Molfino L. Feasibility of using determine-TB LAM test in HIV-infected adults in programmatic conditions. *IAS 2017* 2017.

11 Huerga H, Ferlazzo G, Bevilacqua P, *et al.* Incremental Yield of Including Determine-TB LAM Assay in Diagnostic Algorithms for Hospitalized and Ambulatory HIV-Positive Patients in Kenya. *PLoS One* 2017; 12: e0170976.

12 Meintjes G, Moorhouse M, Carmona S, et al. Adult antiretroviral therapy guidelines 2017. *South Afr J HIV Med* 2017; 18: a776.

13 health-e.org.za. Quicker TB test available for HIV patients in SA. Heal. News. 2017; published online July 27.

14 World Health Organization. Guidelines for intensified tuberculosis case-finding and isoniazid preventive therapy for people living with HIV in resource-constrained settings. *Unpublished* 2011. http://whqlibdoc.who.int/publications/2011/9789241500708_eng.pdf.

15 Wajanga BM, Kalluvya S, Downs JA, Johnson WD, Fitzgerald DW, Peck RN. Universal screening of Tanzanian HIV-infected adult inpatients with the serum cryptococcal antigen to improve diagnosis and reduce mortality: An operational study. *J Int AIDS Soc* 2011; 14: 48.

16 Aoussi EF, Ehui E, Demb JP, *et al.* Cryptoccocal meningitis and HIV in the era of HAART in Cote d'Ivoire. *Med Mal Infect* 2012; 42: 349–54.

17 Grimes RM, Grimes DE. Readiness: The state of the science (or the Lack Thereof). *Curr HIV/AIDS Rep* 2010; 7: 245–52.

18 Rathbun RC, Farmer KC, Lockhart SM, Stephens JR. Validity of a stage of change instrument in assessing medication adherence in indigent patients with HIV infection. *Ann Pharmacother* 2007; 41: 208–15.

19 Naidoo K, Grobler AC, Deghaye N, *et al.* Cost-Effectiveness of Initiating Antiretroviral Therapy at Different Points in TB Treatment in HIV-TB Coinfected Ambulatory Patients in South Africa. *J Acquir Immune Defic Syndr* 2015; 69: 576–84.

15. Appendices

Appendix 1. Screening form
Appendix 2. Informed consent information sheet and consent form
Appendix 3. Case report form (parts 0, 1, and 2)
Appendix 4. TB patient tracing form and follow-up questionnaire
Appendix 5. Outcomes form
Appendix 6. Referral letter

Appendix 7. Recruiting flier
Appendix 8. TB symptom information card
Appendix 9. Adverse event reporting form
Appendix 10. Abnormal lab result form
Appendix 11. Data safety and monitoring plan
