# Supplementary material for: Improved simplified clinical algorithm for identifying patients eligible for immediate initiation of antiretroviral therapy for HIV (SLATE II): protocol for a randomized evaluation
Source: Trials. 2018 Oct 11;19:548. doi: 10.1186/s13063-018-2928-5 (PMC6180640; doi:10.1186/s13063-018-2928-5)
Supplement: Supplementary file 3 — Ethics approvals. (PDF 553 kb) [file 13063_2018_2928_MOESM3_ESM.pdf]

Institutional Review Board
72 E. Concord St., Robinson 4 – Suite 414
Boston, Massachusetts 02118-2307
Tel: 617-638-7207

Title of Study: Randomized Evaluation of a Revised, Simplified Clinical Algorithm for Identifying Patients Eligible for Immediate Initiation of Antiretroviral Therapy for HIV (SLATE II--SIMPLIFIED ALGORITHM FOR TREATMENT ELIGIBILITY II)
IRB Number: H-37010

RE: Initial Review Submission Form
Review Type: Full Board
Action: Approved

Date of Approval: 11/17/2017
Date of Expiration: 11/16/2018

Funding Source: Bill and Melinda Gates Foundation
Award #: OPP1136158

November 17, 2017

Dear Sydney Rosen, MPA:

At the Panel Orange Institutional Review Board (IRB) meeting, chaired by David W. Kaufman, ScD, the Board reviewed the above-referenced submission and determined that it will meet the requirements for approval pending specific changes outlined in the Board's stipulations.

On 11/17/2017, the Chair determined that this study now meets the stipulations set forth by the IRB and is hereby approved. This approval is valid through the expiration date indicated above.

This approval corresponds with the versions of the application and attachments in the electronic system most recently approved as of the date of this letter. The approved version of the attached protocol is 1.1.

Protocol Specific Determinations and Findings
This study has been determined to be not greater than minimal risk under 45 CFR 46. This study will be reviewed as expedited in the future.
No PHI collected, accessed, used or distributed under 45 CFR 164.514.
Written consent will be obtained in accordance with 45 CF 46.116.
Alteration of consent for screening approved under 46.116(d).
Limited- and non-readers may be recruited.

As a principal investigator, you are reminded that you must comply with the responsibilities listed here <http://www.bumc.bu.edu/irb/maintaining-irb-approval/responsibilities-of-the-principal-investigator/>.

Sincerely yours,

Robert Terrano,
Senior IRB Analyst

University of the Witwatersrand, Johannesburg

Human Research Ethics Committee: (Medical)
FWA Registered No IRB 00001223

SECRETARIAT: Suite 189, Private Bag x2600, Houghton 2041, South Africa Tel: +27-11-274 9200 Fax: +27-11-274 9281

05 December 2017

FAXED & COURIERED

Ms N Moyo

Project Manager
Health Economics and Epidemiology Research
39 Empire Road
Parktown
2193

Fax: 086 668 0358

Dear Ms Moyo,

PROTOCOL: SLATE II - RANDOMIZED EVALUATION OF A REVISED, SIMPLIFIED CLINICAL ALGORITHM FOR IDENTIFYING PATIENTS ELIGIBLE FOR IMMEDIATE INITIATION OF ANTIRETROVIRAL THERAPY FOR HIV (SLATE II - SIMPLIFIED ALGORITHM FOR TREATMENT ELIGIBILITY II)

ETHICS REFERENCE NO: 171011

RE : FINAL ETHICS APPROVAL

This is to certify that the above-mentioned trial has been approved by the University of the Witwatersrand, Human Research Ethics Committee (HREC), and the Protocol/Expert Reviewer. Date of Meeting where trial was reviewed: 27 October 2017.

The University of the Witwatersrand, Human Research Ethics Committee Approval Granted for the above mentioned study is valid for five years. Where required by Sponsor to have approval on a more frequent basis it remains the responsibility of the Sponsor and Investigator to apply for continuing review and approval, or for the duration of the Trial.

1. It is the responsibility of the Sponsor and Principal Investigator to ensure, where required, that relevant approvals are in place and compliance with the following is adhered to before a trial may begin:

- If trial is being conducted in Provincial Health facilities: Approval from the Hospital CEO / Clinic Manager / District Research Committee (whichever is applicable) be obtained.
- The study is submitted onto The National Health Research Database (NHRD).
- The relevant approvals are uploaded onto the NHRD system: Ethics Approval, MCC Approval, Hospital CEO / Clinic Manager / District Research Committee Approval.

* A copy of the MCC Approval and/or MCC Notification letter must be submitted to the Ethics Secretariat Office for record purposes (IF MCC APPROVAL / NOTIFICATION IS APPLICABLE).

* The study is conducted according to the protocol submitted to the University of the Witwatersrand, Human Research Ethics Committee. Any amendments to the protocol must first be submitted to the Human Research Ethics Committee for approval.

* During the study, the University of the Witwatersrand, Human Research Ethics Committee is informed immediately of :

- Any Unexpected Serious Adverse Events or Unexpected Adverse Drug Reactions, which, in the Investigator and/or the Sponsor's opinion are suspected to be related to the study drug. (Refer to POL-IEC–001 and SOP-IEC–005, Item 3.4).
- Any data received during the trial which, may cast doubt on the validity of the continuation of the study.

* The University of the Witwatersrand, Human Research Ethics Committee is notified of any decision to discontinue the study and the reason stated.

* The Investigators authorised by this approval participate in this study. Additional Investigators shall be submitted to the University of the Witwatersrand, Human Research Ethics Committee for approval prior to their participation in the study.

* In the event of an authorised Investigator ceasing to participate in the study, the University of the Witwatersrand, Human Research Ethics Committee must be informed and the reason for such cessation given.

2. PRINCIPLES OF INFORMED CONSENT:
* The University of the Witwatersrand, Human Research Ethics Committee requires that in all studies, the Principles of Informed Consent are adhered to. This applies to volunteers as well as patients.

3. PROGRESS REPORTS:
* The University of the Witwatersrand, Human Research Ethics Committee requests that the MCC Progress Reports be submitted twice a year either in March and September or six monthly from start of study to the HREC Secretariat Office - 011 274 9281 and a report of the final results, at the conclusion of the study. (IF APPLICABLE)

4. REIMBURSEMENT TO PATIENTS FOR TRANSPORT:
* The Human Research Ethics Committee: (Medical) is in agreement that reimbursement per visit is according to the Medicines Control Council of SA and that reimbursement should be appropriate according to the situation.

5. TRANSPORT AND STORAGE OF BLOOD AND TISSUE SAMPLES IN SOUTH AFRICA:
* If blood specimens are to be stored for future analysis and is planned that such analysis will be done outside Wits, then the blood must be stored at a facility in South Africa agreed with the relevant IRB, with release of sub-samples only once projects have been approved by the local Research Ethics Committee applicable to where the analysis will be done as well as by the Wits Human Research Ethics Committee: (Medical).

6. GENETIC TESTING:
* The Human Research Ethics Committee: Medical; will not approve open-ended genetic testing as this does not fit the Human Research Ethics Committee criteria.

7. GOOD CLINICAL PRACTICE:
* The South African Department of Health, Medicines Control Council requires Good Clinical Practice (GCP) Training for all Investigators in Clinical Trials, and that GCP training be renewed every three (3) years.

As yet, there are no National Guidelines for the content of GCP courses. Until these are available the Wits Human Research Ethics Committee (Medical) will note courses completed by Investigators without approval of the content of the individual courses.

8. THE SUPPORTING APPROVAL DOCUMENTS ARE ATTACHED:

8.1 Ethics Approval Form signed by the Chairperson of the HREC - Kindly return the copy of the Approval Form signed by the Principal Investigator(s) per fax: 011 274 9281 for our records (this is applicable with the initial Approval).

8.2 List of members present at the HREC meeting held as per INDEPENDENT ETHICS COMMITTEE APPROVAL FORM

9. WE AWAIT YOUR RESPONSES AS REQUESTED: Ensure to have these documents forwarded at the earliest for the HREC records.

* MCC Approval letter and/or letter of Notification before the above study may commence / or where an Amendment may be implemented (IF MCC APPROVAL / NOTIFICATION IS APPLICABLE). It remains the responsibility of the Principal Investigator and/or Sponsor to ensure that the relevant approvals are in place.

* Copy of Independent Ethics Declaration Approval Form signed by the Principal Investigator. (this is applicable with the initial Approval).

* Kindly forward the above to the undersigned at fax: 011 274 9281 at your earliest convenience.

The above has been noted for the Ethics Committee information and records.

KINDLY FORWARD TO THE RELEVANT INVESTIGATORS / CRA / SPONSOR / STUDY CO-ORDINATORS - WHERE APPLICABLE

Regards,

PROF PETER CLEATON-JONES

For and on behalf of the Human Research Ethics Committee: (Medical)

INDEPENDENT ETHICS COMMITTEE APPROVAL FORM

Ethics Reference No.	171011	Date of Meeting	27-Oct-2017
		Recertification Due	26 October 2018 (If applicable)

Principal Investigators:	Dr M Maskew	Investigators:	
	Dr SB Rosen		

Protocol Title: Randomized Evaluation of a Revised, Simplified Clinical Algorithm for Identifying Patients Eligible for Immediate Initiation of Antiretroviral Therapy for HIV (SLATE II - SIMPLIFIED ALGORITHM FOR TREATMENT ELIGIBILITY II)

	DOCUMENTS REVIEWED	Tick As Appropriate	Yes	No
Protocol Number	SLATE II	Date: 05-Oct-2017		
Protocol	SLATE II Research Protocol - Version 1.0	Date: 05-Oct-2017	☑	☐
Investigator's Brochure	N/A - Version: N/A - Dated:		☑	☐
Subject Information/Consent Form	Research Information Form - Version: 1.2 - Dated: 16 Oct 2017		☑	☐
	Information for Patients starting ART with TB Symptoms - Version: - - Dated:		☑	☐
Advertisements	Advertisement: "Join the SLATE II Study" - Version: - - Dated:		☑	☐
Questionnaires	SLATE II ID Form (CRF Part 0) - Version: - - Dated: 05 Oct 2017		☑	☐
	SLATE II Screening Form - Version: - - Dated: 05 Oct 2017		☑	☐
	SLATE II CRF Lab Result and Tracing Form - Version: - - Dated: 05 Oct 2017		☑	☐
	SLATE II Referral Letter - Version: - - Dated: 06 Oct 2017		☑	☐
	SLATE II CRF Adverse Event Reporting Form - Version: - - Dated: 05 Oct 2017		☑	☐
	SLATE II CRF Outcomes Form - Version: - - Dated: 06 Oct 2017		☑	☐
	SLATE II TB tracing and questionnaire - Version: - - Dated: 06 Oct 2017		☑	☐
	SLATE II CRF Part 2 - Version: - - Dated: 06 Oct 2017		☑	☐
	SLATE II CRF Part 1 - Version: - - Dated: 06 Oct 2017		☑	☐
Insurance/Compensation	N/A	Valid From To:	☐	☑
Synopsis of Study/Trial Summary	SIMPLIFIED ALGORITHM FOR TREATMENT ELIGIBILITY II		☑	☐
Other Documentation	Protocol Synopsis - Dated: 05 Oct 2017		☑	☐
	NHREC Trial Application ID#: 4852 - Dated: 05 Oct 2017		☑	☐
	ClinicalTrials.gov Protocol Registration and Results System - Dated: 06 Oct 2017		☑	☐
	HREC Application Form - Dated: 06 Oct 2017		☑	☐
	Subcontract Scope of Work - Dated:		☑	☐
Relevant Trial Hospital/(s)	Jabulane Dumane Community Health Centre		☑	☐
	OR Tambo Clinic		☑	☐
	Alexandra Health Clinic		☑	☐
	Health Economics and Epidemiology Research Office (HERO)		☑	☐
Syndicate and/or Research Unit	Health Economics and Epidemiology Research Office (HERO)		☑	☐

DETAILS OF COMMITTEE	
Name	University of the Witwatersrand Human Research Ethics Committee: (Medical)
Address	Research Office, Senate House<br>University of the Witwatersrand, 1 Jan Smuts Avenue, BRAAMFONTEIN, Johannesburg, 2000

DETAILS OF MEETING	Yes	No
Is the Investigator a member of the committee ?	☐	☑
If "Yes" did he/she vote ?	☐	☑
Is the Committee organised and operated according to applicable laws and regulations together with ?	☑	☐
Local GCP requirements ?	☑	☐
ICH GCP requirements ?	☑	☐
FDA GCP requirements ? FWA Registered No. IRB00001223	☑	☐
Progress reports required either in March and September or six monthly from start of study ?	☑	☐

DECISION ON APPROVAL : is approval given to conduct the trial ?		Tick As Appropriate
Yes - with no conditions		☑
Remarks :	Our expectation is that our requirements are fulfulled once a company has submitted and uploaded relevant approvals onto NHRD.	
Yes - with conditions		☐
Specify conditions :		
No		☐
Specify reasons		

INDEPENDENT ETHICS COMMITTEE APPROVAL FORM

SIGNATURES			
I confirm that the details on this form are correct:			Date
**Name:** **Prof PE Cleaton-Jones** **Chair / Deputy Chair of Committee**	**Signature:**	[signature]	**05 December 2017**

DECLARATION OF INVESTIGATOR/(S)

To be completed and ONE COPY returned to the Secretariat for the HREC at Wits Health Consortium, 8 Blackwood Avenue, Parktown, 2193 or Fax To: 011 274-9281

I/We fully understand the conditions under which I am/we are authorised to carry out and complete the above-mentioned research and I/we agree to ensure full compliance with these conditions. Should any amendment, alteration or departure be contemplated from the research procedure methodology or manner of execution, I/we will communicate with the Chairman of the Human Research Ethics Committee: (Medical) for approval prior to acting on any of the above mentioned proposed amendments, alterations or departures. I am/we are fully aware that any unauthosised amendment, alteration or departure as above will amount to misconduct and may lead to the institution of disciplinary procedures.
Any approval given by the HREC is conditional upon consent being obtained by the Investigator/s from the Superintendent (or equivalent official) of the Hospital, Clinic or Institution in which the research is, in part or full, to take place.
The Chairman may of course at his discretion place the matter before the full Committee.

DATE:______________________ SIGNATURE:____________________________ NAME:____________________________

PROTOCOL NUMBER **SLATE II** ETHICS REF.: **171011**

Date Printed 05 December 2017

HUMAN RESEARCH ETHICS COMMITTEE MEMBERS: (MEDICAL)
UNIVERSITY OF THE WITWATERSRAND

Attendance Register for the Ethics Meeting held on 27 October 2017 from 11:00 - 15:00
Venue: EXECUTIVE COMMITTEE ROOM, Ground Floor, Phillip V Tobias Building, Cnr York Road & 29 Princess of Wales Terrace

AFFILIATED TO THE UNIVERSITY OF THE WITWATERSRAND

Surname	Initials	Title	Discipline/s	Academic Qualifications	Gender	Present
Adam	Y	Dr	Obstetrics & Gynaecology	MB BCh; FCOG	F	Absent
Cleaton-Jones	PE	Prof	Biomedical Ethics	BDS; MB BCh; PhD; DA (SA); DTM&H; DSc (Dent), FCD (SA) DPH; PhD Hon Causa, MASSAfr	M	Present
Conradie	FM	Dr	Infectious Diseases/HIV/TB	MB BCh; DTM&H; MSc; Dip HIV Man	F	Present
Cooper	PA	Prof	Paediatrics	MB BCh, PhD, DCH (SA), FCPaeds (SA)	M	Present
Dhai	A	Prof	Biomedical Ethics	MB ChB; FCOG; LLM; PGDipIntResEthics	F	Present
Donde	B	Prof	Radiation Oncology	MB BCh, MMed Rad (T)	M	Absent
Etheredge	H	Dr	Biomedical Ethics	MSc Med, BA; PhD	F	Present
Feldman	C	Prof	Pulmonology	MB BCh, PhD, DSc, FCP (SA), FRCP	M	Present
Gerrand	P	Dr	Social Work	PhD (Social Work)	F	Present
Lowman	W	Dr	Clinical Microbiology	MB BCh, MMed, FC Path (SA) Micro	M	Absent
Lownie	MA	Prof	Maxillo-Facial & Oral Surgery	BDS, BA (Hons), DipMFOS, FCMFOS(SA), MEd	F	Absent
Menezes	CN	Prof	Internal Medicine	MD, MMed (Int Med), Dip HIV Mang (SA), DTM&H, FCP (SA), Cert ID (SA), PhD	M	Absent
Naidoo	S	Prof	Public Health	MB BCh, DMTH, DHSM, DOH, MMED	M	Absent
Naran	NH	Dr	Chemical Pathology	PhD	M	Present
Penn	C	Prof	Speech Pathology	BA (Sp&HTh), PhD, CCC SL-P, OMS	F	Absent
Penny	C	Prof	Internal Medicine	BSc Hons, PhD	M	Present
Ross	M	Prof	Public Health	MB ChB, FFCH(SA), FOM(UK)	F	Absent
Sanne	IM	Prof	Infectious Diseases/HIV/TB	MB BCh, FCP (SA), DTM&H; MMed & PhD	M	Absent
Smith	C	Prof	Psychiatry	BA, BA (Hons), M.A (Clin.Psych), PhD	F	Present
Stewart	A	Prof	Physiotherapy	BSc (Physio), MSc, PhD, DPE	F	Absent
Szabo	CP	Prof	Psychiatry	MB BCh, MMed, MScMed, PhD; FCPsych(SA)	M	Absent
Thom	RGM	Prof	Psychiatry	MB ChB, DCH, FCPsych, PhD	F	Absent
Tsotsi	NM	Dr		BDS; MPH; MSc Med; PGDipInt ResEthics	F	Present
Velaphi	S	Prof	Paediatrics	MB BCh, FCPaeds, MMed	M	Present
Warria	A	Dr	Social Work	D Litt et Phil (SWK)	F	Absent
Willem	P	Dr	Human Genetics	MD, PhD	F	Absent
Woodiwiss	AJ	Prof	Cardiovascular Pathophysiology	BSc Physiotherapy, BSc, MSc, PhD	F	Absent

NOT AFFILIATED TO THE UNIVERSITY OF THE WITWATERSRAND

Surname	Initials	Title	Discipline/s	Academic Qualifications	Gender	Present
Barnabas	N	Ms	Civil Society Liaison Officer	Community Liaison Manager	F	Present
Burns	I	Mr	Community Representative	MA (Hons)		Present
Egan	A	Father	Theology	BA (Hons), MA, MDiv, STL, PhD	M	Present
Guidozzi	Y	Adv	Lawyer	BSc (Nurs), LLB, MBA	F	Present
Ikalafeng	B	Dr	Governance	BSc (Hons), MSc, PhD	F	Absent
Langley	G	Prof	Nursing	MSc (Nursing), PhD, MPhil (Ethics)	F	Present

Mokhachane	M	Dr	Community Representative	MB BCh, FCP (Paeds) SA, MMed, Neonatology (SA)	F	Absent
Paruk	F	Prof	Anaesthesia	MB ChB, FCOG(SA), Crit Care(SA), PhD	F	Absent
Peter	JR	Adv	Lawyer	BCom; LLB; LLM	M	Absent
Van Gelderen	CJ	Prof	Obstetrics & Gynaecology	MB BCh, FRCOG, FCOG(SA)	M	Absent

RETIRED MEMBER OCCASIONALLY CO-OPTED FOR SURGICAL OPINION ON A PROJECT

Oettle	GJ	Prof	Surgery	BSc (Hons), MB BCh, FRCS	M

Note 1: This committee has been in continuous operation since October 1966

Note 2: The large committee size is to ensure a good attendance at meetings

Note 3: A Quorum is 13 members according to the 33% of members on a committee with more than 15 members as required by SA National Guidelines (ref 2 below)

This is to certify that the Human Research Ethics Committee: (Medical) of the University of the Witwatersrand operates according to the following guidelines of good clinical practice:

1. **ICH Harmonised Tripartite Guideline for Good Clinical Practice.**
2. **SA National Department of Health 2006 Guidelines for Good Practice in the Conduct of Clinical Trials with Human Participants in South Africa (2006).**
3. **Declaration of Helsinki 2013.**

The Committee's United States Federal Wide Assurance details are:

1. **Country code SF.**
2. **FWA Number: FWA00000715.**
3. **University of the Witwatersrand: IORG0000862.**
4. **Human Research Ethics Committee: (Medical): IRB00001223.**
