# Supplementary material for: Improved simplified clinical algorithm for identifying patients eligible for immediate initiation of antiretroviral therapy for HIV (SLATE II): protocol for a randomized evaluation
Source: Trials. 2018 Oct 11;19:548. doi: 10.1186/s13063-018-2928-5 (PMC6180640; doi:10.1186/s13063-018-2928-5)
Supplement: Supplementary file 4 — Informed consent form. (PDF 134 kb) [file 13063_2018_2928_MOESM4_ESM.pdf]

RESEARCH INFORMATION FORM

Title of Project: Performance Evaluation of a Revised Simplified Clinical Algorithm for Identifying Patients Eligible for Immediate Initiation of Antiretroviral Therapy for HIV (SLATE II)

Principal Investigator: Sydney Rosen

Study Number:	
Study Title:	**Randomized Evaluation of a Revised, Simplified Clinical Algorithm for Identifying Patients Eligible for Immediate Initiation of Antiretroviral Therapy for HIV (SLATE II)**
Sponsor:	Bill & Melinda Gates Foundation
Investigators:	Prof Sydney Rosen (Boston University); Dr. Mhairi Maskew (Wits University)
Telephone numbers:	+ 1-617-414-1273 (Prof Rosen, U.S.); +27 21 794 8856 (Dr. Maskew, South Africa)

Good day. My name is _______________, and I am a Study Assistant at the Health Economics and Epidemiology Research Office at the University of the Witwatersrand. I would like to invite you to consider participating in a research study entitled “Randomized Evaluation of a Revised, Simplified Clinical Algorithm for Identifying Patients Eligible for Immediate Initiation of Antiretroviral Therapy for HIV (SLATE II)”.

What Is this Study About?

Boston University in the United States and Wits University in South Africa are conducting a research study for patients who have HIV but have not yet started HIV treatment, called antiretroviral therapy or ART. The study will try to find out whether offering patients the chance to start ART today, using a simple set of questions and a brief physical examination, is a good way to encourage earlier treatment initiation and reduce the number of patients who don’t come back to the clinic to start treatment. If the new process works well, then it could be adopted by all the clinics in the country to help patients get onto ART, improve outcomes on ART, and reduce the overall number of times patients have to come to the clinic, which could reduce costs for both patients and clinics. This is the second study we are doing to answer this question, as we have made some improvements since the first study (called SLATE I) and want to see if the new process works better.

To conduct this research study, we are inviting adult, non-pregnant patients in this clinic who have HIV but have not yet started ART to participate in the study. If you participate, you will be assigned at random (by chance) to one of two groups. One group (called the Standard Group) will follow the clinic’s usual HIV treatment initiation schedule. The other group (called the Intervention Group) will be offered the chance to start HIV treatment at the end of today’s visit. If you choose to participate, you will be asked to participate in the study starting today and continuing for up to 16 months from today, though you will probably not have any contact with the study team after today.

This study is being conducted by Professor Sydney Rosen from Boston University and Dr. Mhairi Maskew in South Africa. Other investigators involved in the study are Professor Matthew Fox and Dr.

BMC and BU Medical Campus IRB
IRB NUMBER: H-37010
IRB APPROVAL DATE: 11/17/2017

Alana Brennan from Boston University and Ms. Lungsile Vezi from Wits University. This study is being sponsored by the Bill & Melinda Gates Foundation in the United States.

Taking part in this study is voluntary. You can choose not to take part and if you join, you may quit at any time. If you decide not to participate, you will receive the same care from this clinic that you would have received otherwise.

What is the Purpose of this Study?

It is very important for patients who are infected with HIV to start treatment as quickly as possible. By starting treatment earlier, patients will become less sick, the treatment will work better, and they will need less other medical care. Unfortunately, many patients do not start treatment when it is recommended, even if they know they can.

While there are many reasons that patients do not start as early as they should, one reason is that starting treatment has required several visits to the clinic, with delays of up to several weeks in between. A large number of patients do not come back for all the visits, and therefore don't start treatment when they should. Requiring patients to make several visits to the clinic in order to start HIV treatment also creates a burden for the clinic, leading to long waiting times for patients and taking time from clinic staff that they could use to help more patients.

With many new patients eligible for ART and limited space at clinics, new ways to organize HIV treatment initiation are needed. It is important that whatever new approach is adopted be safe and effective, so that patients who should start treatment immediately (today) can do so, while those who should not are referred for whatever other care they need. It is likely that most patients will be able to start treatment immediately, but some will need additional laboratory tests, different care, or more time to make the decision that they are ready. The South African National Department of Health has asked clinics to offer same-day treatment initiation to patients who are ready, but it has not explained exactly what procedures should be used.

The purpose of this study is to compare two procedures for starting treatment. One procedure, for the Standard Group, is the normal one that this clinic uses, which could mean starting ART today or could require more clinic visits after today. The other procedure, for the Intervention Group, uses a set of questions and a short physical examination to collect the information needed to see if a patient is ready to start ART today. Patients who are ready will get their first supply of medications today, while patients who are not ready will receive standard clinic care. The two procedures use exactly the same medications and do not change the treatment itself in any way. They are mainly different in the exact steps they take and the timing of those steps. By comparing patients who follow the two different procedures, the study will determine whether the study's approach to providing immediate treatment initiation today is a good way to reduce delays and encourage earlier treatment, whether it is convenient for patients and for the clinic, and how much it costs compared to the standard schedule.

What Happens in this Research Study?

This study will take place at three clinics in South Africa. You will be one of approximately 600 patients to be asked to participate in this study. Patients in this study must be at least 18 years old, not pregnant, have HIV, and not yet have started HIV treatment. If you participate in the study, it will take about 30-90 minutes of your time today.

BMC and BU Medical Campus IRB
IRB NUMBER: H-37010
IRB APPROVAL DATE: 11/17/2017

RESEARCH INFORMATION FORM

Title of Project: Performance Evaluation of a Revised Simplified Clinical Algorithm for Identifying Patients Eligible for Immediate Initiation of Antiretroviral Therapy for HIV (SLATE II)

Principal Investigator: Sydney Rosen

For all patients in the study:

After you agree to be in the study, if you are a woman, the first step is to have a pregnancy test, in case you are pregnant and not aware of it. If you are found to be pregnant, you will not be able to continue with the study, as the study is only for non-pregnant patients. If you are pregnant, you should continue with antenatal and HIV care at the clinic under the usual procedures.

Then, for all patients, the study assistant will first ask you for your name, date of birth, and details of how to contact you if necessary. You will then be asked some questions about yourself and your household (such as how many other people you live with and whether any of them know you have HIV), your activities (such as whether you have a job or not), how much it costs you to come to this clinic (transport fare and other costs), your opinions about how fast HIV treatment should be started, and whether you have any of the common symptoms of tuberculosis (TB). You will not have to answer any question you do not want to answer.

After answering these questions, you will be assigned at random (by chance) to either the Intervention Group or the Standard Group.

For patients in the Intervention Group:

If you are in the Intervention Group, the study assistant will introduce you to the study clinician (a nurse or clinical officer). The study clinician will then carry out five steps:

- Ask you questions about whether you have any symptoms of conditions that might be related to HIV, including TB.
- Ask you questions about your health in the past and treatment you might have received in the past.
- Do a short physical exam to see if you have any conditions that might require additional care.
- Ask you questions and talk with you about whether you are ready to start ART today.
- Draw blood for a CD4 count and other laboratory tests that are routinely done at this clinic and collect a sputum sample for a TB test for all patients. The amount of blood to be drawn will be up to 20 ml (equal to a maximum of 4 teaspoons).

Most of the study procedures involve answering questions. Having your blood drawn from your arm may cause you temporary discomfort or pain and may leave a temporary bruise or swelling at the point where the blood is taken. Some patients may be uncomfortable providing a urine sample and/or a sputum sample or having a physical examination. None of these procedures, however, are different from what is asked under standard care.

If you have symptoms of TB, like coughing or a fever, the nurse will collect two other types of

BMC and BU Medical Campus IRB
IRB NUMBER: H-37010
IRB APPROVAL DATE: 11/17/2017

information. First, she will ask you for a urine sample in order to do a test called LAM, which is short for lipoarabinomannan antigen of mycobacteria. (Women who have already provided a sample for a pregnancy test will not have to do it again.) LAM is one way to diagnose TB in patients with more advanced HIV infection. Second, she will ask you more detailed questions about your symptoms (for example, for how long have you been coughing). The nurse will use the results of the LAM test, questions, and physical exam to decide if you should wait for the results of the regular TB test before you can start ART or can instead start immediately.

Each of these steps will take just a few minutes to complete. The study clinician will then look at all the information collected about you and tell you whether you can start ART immediately or should instead have a regular clinic visit to follow up on some of the information collected. For example, if you have symptoms of serious illness, you will need to see a nurse or doctor at the clinic before you can start ART.

If you can start ART immediately, the study nurse will write a prescription for your first supply of ARV medications, and the study assistant will help you get the prescription filled by the clinic's pharmacy. The study assistant will also help you make an appointment for your next regular clinic visit. It is very important that you make and keep this appointment, which should include support for ART adherence as well as a medication refill when needed.

If you cannot start ART immediately, for example because additional care is needed or you would like to have more time before you start, the study assistant or study clinician will take you back to the appropriate location for the service you need in the clinic. The study clinician will give you a letter that you may give to the clinic nurse or clinical officer who sees you, to tell that person why the study clinician thought additional care was needed. You do not have to give the letter to the nurse or medical officer if you do not want to. You will then follow the standard procedures at the clinic for starting ART.

If you do start ART immediately (today), and are then found to have TB, the study staff will do their best to contact you tomorrow, or as soon as they learn the results of the TB test. They will first attempt to call you on the mobile phone number(s) you have provided. If they cannot reach you by phone on the day the test results are available (most likely tomorrow), either a member of the study staff or a community health worker or WBOT worker from the clinic will visit your home. We will only speak to you (not another person) during the calls and visit. If we do not find you, we will text the message "SLATE" to your mobile phone number. It is important that you start TB treatment immediately if you have TB, both to treat your TB and to ensure that the HIV treatment does not cause any other illness, as it can in patients with active TB. For this reason, it is very important that you provide correct contact details when asked.

If you do start ART immediately (today), and are then found to have cryptococcal infection, the study staff will alert the clinic staff to this diagnosis so that the clinic can trace you using their regular tracing procedures.

After this, you will not have any more direct (face-to-face) contact with the study researchers unless you have TB. For patients without TB, during the rest of your participation in the study the researchers will collect data from your regular clinic file only.

<u>For patients in the Standard Group</u>:

If you are in the Standard Group, all your care will be the normal care provided by the clinic. The study assistant will take you back to your original place in the queue or to the front of the queue if

BMC and BU Medical Campus IRB
IRB NUMBER: H-37010
IRB APPROVAL DATE: 11/17/2017

your original place has already been called, so that you do not have to start at the back of the queue again before the next step in your regular clinic visit. You will not have any more direct (face-to-face) contact with the study researchers if you are in the Standard Group. You will be given a copy of a form indicating the results of the TB symptom questions that were asked in the questionnaire, so that you can give this to the clinic nurse you see.

For all patients with a positive TB test:

If you test positive for TB in either study group, we will ask you to stop by our study rooms at your next regular clinic visit (usually 2-4 weeks from now) to answer a few short questions about your health since you enrolled in the study. This will take about 10 minutes to complete.

For all patients after today:

For the rest of your participation in the study, the researchers will collect data from your regular clinic file only. This will include information from the clinic's register books, files, and other regular sources of data. The clinic file will be used to find out whether and how often you come back to the clinic, when you start ART, what drugs and tests you receive, and what the results of your treatment are. The researchers will collect this information at several time points after you enroll in the study, up to a maximum of 16 months from now. You will not have any contact with the study staff again, after today, only with the regular clinic staff when you come for your HIV care visits. Collecting this information will have no effect on the care you receive from this clinic.

Are There Risks or Discomforts from Participating?

The care and treatment you will receive in this study are almost the same as you would receive otherwise. If you are in the Intervention group, the procedures followed before you start treatment will be different from standard care, but the treatment itself will be exactly the same.

However, there are some risks to you if you decide to participate. The research team will do everything possible to decrease these risks, which are:

- Starting patients with active TB patients on ART, before treating for TB, can cause IRIS (immune reconstitution inflammatory syndrome). If you develop IRIS, you may have a new cough, fever, night sweats, or weight loss. If you already have these symptoms, they may become worse. This is why standard care requires TB test results for anyone with TB symptoms before starting ART. IRIS is more common in very sick patients, and most patients who start ART today in the intervention group will not be very sick, as we will refer you for additional care if you are very sick. The study takes several steps to find patients who have active TB, using the symptom questions, LAM test, and physical exam.

BMC and BU Medical Campus IRB
IRB NUMBER: H-37010
IRB APPROVAL DATE: 11/17/2017

It is still possible, however, that a patient who starts ART immediately will have TB and will develop IRIS. To reduce this risk, study staff will monitor laboratory testing results carefully, and any patient who was initiated on ART the previous day and has a positive TB test after that will immediately be traced by the study staff, informed of the test result, and asked to return to the clinic for further assessment and TB treatment. We will also give you an information card that reminds you to return to the clinic if your TB symptoms worsen or new ones develop, as could happen with IRIS.

- Some patients who start ART immediately may find that, after some days or weeks on treatment, they were not personally ready to be on treatment after all. These patients may stop taking their medications. It is possible that this would not have happened if they had started treatment more slowly, following the usual schedule. To avoid this, the study staff will be sure that you have time to ask questions and discuss whether you are ready to start immediately and what to expect after you start. The clinic's staff will also be available after you start treatment to answer questions or help you with any concerns.

- For patients who have just learned that they are HIV-positive, emotional distress is common. Because for some patients the study procedures will take place immediately after the HIV test, some participants may find that the study makes them feel more distressed. The study staff and clinic staff will make every effort to reduce your distress. Please inform any member of the staff if you feel that you do not want to remain in the clinic to participate in the study or would like to speak individually with someone on the study or clinic staff.

- Pregnant women should not enroll in this study, as the research is about non-pregnant adults. If you are not pregnant today but you find out later that you are, we will withdraw you from the study. We will not use your data for the study and will destroy all data that we have collected on you so far.

- In addition, there may be unknown risks or discomforts involved. Study staff will update you in a timely way on any new information that may affect your health, welfare, or decision to stay in this study.

Are There Potential Benefits from Participating?

If you are in the Intervention Group, the study may provide a direct benefit to you by allowing you start ART immediately, without having to make additional visits to the clinic. There will not be any direct benefits to participants who are in the Standard Group. However, your participation may help the researchers find out whether a simplified process for starting HIV treatment is just as good as what is currently done. This may in turn make starting HIV treatment easier for many other HIV patients, as well as saving clinic resources so that the clinic staff can provide better care to everyone.

What Other Choices Do I Have?

Your alternative is not to participate in this study.

Are There Any Costs or Payments to Me?

There are no costs to you for participating in this study. All of the care offered for participants in the study is free, and ART is free at this clinic. If you consent to participate in this study and complete study procedures, you will receive compensation equivalent to R150 in the form of a shopping

BMC and BU Medical Campus IRB
IRB NUMBER: H-37010
IRB APPROVAL DATE: 11/17/2017

voucher to thank you for your time and any inconvenience of being in the study, including travel and refreshments.

How Will My Information Be Protected?

The study researchers will do everything they can to protect your confidentiality during this study. During the study, your name, date of birth, and file numbers at this clinic will be collected. This will allow the researchers to match up different types of information about you, such as the questionnaire answers and your clinic file. Before any of this information is used in the study, however, the information that identifies you, such as your name, will be replaced by a random study identification number that no one else can use to identify you. A list of study participants' names, dates of birth, national identity numbers, and file numbers will be kept in a secure, confidential location, so that only the study researchers can access it. For conducting the research, only your study identification number will be used. Your name or other information that would allow someone outside the study to identify you will never be used in study publications or reports.

In addition to the researchers, the ethics committees of the University of the Witwatersrand Human Research Ethics Committee (Medical) and Boston University and the Office of Human Subject Protection in the U.S. Department of Health and Human Services are authorized to review study data. A description of this study will be available on http://www.ClinicalTrials.gov. This web site will not include information that can identify you. At most, the web site will include a summary of the results. You can search this web site at any time.

Participant's Rights

By consenting to participate in this study, you do not waive any of your legal rights. Giving consent means that you have heard or read the information about this study and that you agree to participate. You will be given a copy of this form to keep.

If at any time you withdraw from this study you will not suffer any penalty or lose any benefits to which you are entitled.

The researchers or a member of the research team will try to answer all of your questions. If you have questions or concerns at any time, or if you need to report an injury while participating in this research, you may contact the investigators listed above at the beginning of this form.

This clinical study protocol has been submitted to the University of the Witwatersrand, Human Research Ethics Committee (HREC) and written approval has been granted by that committee.
The study has been structured in accordance with the Declaration of Helsinki (last updated: October 2013), which deals with the recommendations guiding doctors in biomedical research involving human participants. A copy may be obtained from me should you wish to review it.

BMC and BU Medical Campus IRB
IRB NUMBER: H-37010
IRB APPROVAL DATE: 11/17/2017

If you want any information regarding your rights as a research participant, or complaints regarding the research study, you may contact Prof Cleaton-Jones, Chairperson of the University of the Witwatersrand, Human Research Ethics Committee (HREC), which is an independent committee established to help protect the rights of research participants, at +27-11-717-2301.

You may also contact the Office of the Institutional Review Board of Boston University Medical Center in the United States, tel. +1-617-638-7207.

Right to Refuse or Withdraw

Taking part in this study is voluntary. You have the right to refuse to take part in this study. If you decide to be in the study and then change your mind, you can withdraw from the research. Your participation is completely up to you. Your decision will not affect your being able to get health care at this clinic, payment for your health care, your enrollment in any medical aid or insurance plan, or other health benefits you can get.

If you choose to take part, you have the right to stop at any time. If there are any new findings during the study that may affect whether you want to continue to take part, you will be told about them as soon as possible.

The investigator may decide to discontinue your participation without your permission because he/she may decide that staying in the study will be bad for you, or the sponsor may stop the study.

Signatures

Signing this consent form indicates that you have read this consent form (or have had it read to you), that your questions have been answered to your satisfaction, and that you voluntarily agree to participate in this research study. You will receive a copy of this consent form to keep.

________________________________	___________________________	_____________	______
Participant (Signature or Thumbprint)	(Printed Name)	Date	Time
________________________________	___________________________	_____________	______
Person Obtaining Consent (Signature)	(Printed Name)	Date	Time
________________________________	___________________________	_____________	______
Witness* (Signature)	(Printed Name)	Date	Time

*Witness signature required if patient provides mark or thumbprint rather than signature

BMC and BU Medical Campus IRB
IRB NUMBER: H-37010
IRB APPROVAL DATE: 11/17/2017
